# A novel representation of RNA secondary structure based on element-contact graphs

**DOI:** 10.1186/1471-2105-9-188

**Published:** 2008-04-11

**Authors:** Wenjie Shu, Xiaochen Bo, Zhiqiang Zheng, Shengqi Wang

**Affiliations:** 1 Beijing Institute of Radiation Medicine, Beijing 100850, China; 2 College of Electro-Mechanic and Automation, National University of Defense Technology, Changsha, Hunan 410073, China

## Abstract

**Background:**

Depending on their specific structures, noncoding RNAs (ncRNAs) play important roles in many biological processes. Interest in developing new topological indices based on RNA graphs has been revived in recent years, as such indices can be used to compare, identify and classify RNAs. Although the topological indices presented before characterize the main topological features of RNA secondary structures, information on RNA structural details is ignored to some degree. Therefore, it is necessity to identify topological features with low degeneracy based on complete and fine-grained RNA graphical representations.

**Results:**

In this study, we present a complete and fine scheme for RNA graph representation as a new basis for constructing RNA topological indices. We propose a combination of three vertex-weighted element-contact graphs (ECGs) to describe the RNA element details and their adjacent patterns in RNA secondary structure. Both the stem and loop topologies are encoded completely in the ECGs. The relationship among the three typical topological index families defined by their ECGs and RNA secondary structures was investigated from a dataset of 6,305 ncRNAs. The applicability of topological indices is illustrated by three application case studies. Based on the applied small dataset, we find that the topological indices can distinguish true pre-miRNAs from pseudo pre-miRNAs with about 96% accuracy, and can cluster known types of ncRNAs with about 98% accuracy, respectively.

**Conclusion:**

The results indicate that the topological indices can characterize the details of RNA structures and may have a potential role in identifying and classifying ncRNAs. Moreover, these indices may lead to a new approach for discovering novel ncRNAs. However, further research is needed to fully resolve the challenging problem of predicting and classifying noncoding RNAs.

## Background

Recent years have witnessed an explosive growth in RNA research, as numerous new noncoding RNAs (ncRNAs) have been discovered [1,2], and rich information has been revealed in the various relationships between their structures and cellular functions [3]. It is increasingly evident that RNAs play important roles, far beyond transferring genetic information from DNA to protein. Exploring the structural diversity of the RNA population constitutes a central goal in RNomics [4], which requires new computational methods for the comparison, identification and classification of RNA.

As there remain many difficulties in predicting three-dimensional RNA structure, secondary structures are typically used as a basis for researching RNA conformation. RNA secondary structure can be viewed as a combination of basic structural elements, also known as stems, hairpin loops, bulge loops, interior loops, multiple loops and external loops (the latter five categories are referred to collectively as 'loops'). Mathematical representations of RNA secondary structure are of great importance. Some approaches for deducing these structures have been proposed as planar graphs [5-9]. Among these RNA representations [5-7,9-11] is the homeomorphically irreducible tree (HIT) [10], which contains most of the RNA molecule's original structural information. Each HIT node corresponds to a structural element weighted by its 'size'. The stem elements are weighted by the number of contained base pairs, while the loop elements are weighted by their lengths. The topological nature of a HIT is a vertex-weighted and vertex-colored tree graph, in which the stem and loop vertices are color-coded. Most of the other RNA graphs give unequal prominence to stems and loops in the secondary RNA structures, that is, the stem regions are always represented as adjacent relationships between loop vertices and cannot be reflected directly in the matrix representations and numerical descriptors. The rationality of this abstraction may depend on the opinion that single-stranded regions play important roles in RNA-RNA, RNA-DNA and RNA-protein interactions. However, some studies have revealed that stem regions are of the same importance as loop regions. For example, recent studies show that stem regions in precursors of miRNAs are indispensable for miRNA biogenesis [12-15]. Considering that stems and loops are biochemically different, an ideal RNA graphical representation should distinguish these two element types.

Graphical representation of RNA secondary structure provides the basis for the construction of topological indices. Topological indices are numeric parameters associated with patterns of connectivity among vertices, reflecting the intrinsic nature of a graph. In computational compound design, topological indices have been successfully employed in many applications such as QSAR (quantitative structure-activity relationships) and QSPR (or quantitative structure-property relationships) [16]. For RNA-related research, topological indices based on RNA graphics provide simple solutions for structure comparison, classification and enumeration [5-7,17-20], and are gaining increasing acceptance in the scientific community. In recent innovative works, Schlick *et al *successfully used topological indices from tree and dual graphs to explore the repertoire of RNA secondary motifs [8,9,21,22], and further uncovered structural diversity in random sequence pools [23].

However, it is difficult to construct topological indices to characterize the colors of the HIT-like fine-grained RNA graphs, because the node colors encoded in the polarity of items in the topological index definition renders the range of topological indices uncontrollable, even unmanageable in extreme cases. On the other hand, ignoring the length of the loop and stem regions can lead to index degeneracy. The RNA topological indices presented herein focused mainly on molecular connectivity descriptions. Although these indices reflect some significant aspect of RNA structure and show good performance in distinguishing between different structural patterns, they may not be appropriate for characterizing structural details. As a consequence, RNAs with different structures may share the same index value. The latent risk of high degeneracy derives mainly from the coarse-grained abstraction in RNA graph construction. Additionally, even for connectivity, no single index is sufficient. A numerical descriptor derived from the spectrum of the Laplacian matrix of the RNA graph, which has been widely used recently [8,9,17-21,24], cannot uniquely determine graph topology when the vertex number is greater than five [25].

In this study, we present a complete and fine scheme to represent RNA molecules graphically. These representations will facilitate the exploration of the numerous detailed facets of each RNA element and their combined patterns in creating RNA secondary structures. Herein we introduce three typical examples of information-rich topological indices that are based on our novel graph representations to characterize the RNA secondary structure. The involvement of the numerical range, distribution and intercorrelations of these indices for their possible rendering of useful RNA topologies are presented, and the applicability of these indices is illustrated by three case studies.

## Results

### Statistical properties of topological indices

#### Numerical range and distribution of topological indices

The utility of topological indices depends mostly on the mathematical properties of the indices, such as where and how an index maps RNA molecules from structural space to numerical space. Herein, we provide a detailed analysis on the relationship between the topological indices and the RNA secondary structure on a dataset of 6,305 ncRNA sequences (listed in Table 1). We calculated the values of the topological indices for these 6,305 ncRNAs, and try to find their connections with RNA secondary structures. In addition, we attempted to reveal the connections among the topological indices and the RNA molecule lengths, free energies and GC contents.

**Table 1:** Dataset of ncRNA sequences. A dataset of 6,305 ncRNAs taken from different Database are selected as representatives of the RNA world. These 6,305 ncRNA sequences are classified into two classes: one class covers five kinds of ncRNAs with known structures, and the other class is made up of six kinds of ncRNAs with predicted structures by Vienna RNA package.

Category	Number	Length (nt)			dG (Kcal/mol)	GC%
				
		Mean ± SD	Min	Max		
**The sequences with known structures**						

5S ^(1)^	147	121 ± 3	113	135	-49.97 ± 10.08	0.58 ± 0.05
16S ^(1)^	647	1532 ± 284	612	2741	-556.15 ± 156.85	0.49 ± 0.08
Intron ^(1)^	144	615 ± 418	210	2630	-191.53 ± 99.46	0.46 ± 0.11
RNase P ^(2)^	466	332 ± 49	189	486	-139.83 ± 35.4	0.57 ± 0.09
tRNA ^(3)^	1272	76 ± 5	56	94	-29.28 ± 5.31	0.58 ± 0.06

**The sequences with predicted structures**						

tmRNA ^(4)^	140	359 ± 30	251	423	-117.44 ± 25.45	0.47 ± 0.09
5.8S ^(4)^	1168	146 ± 27	29	180	-41.33 ± 11.99	0.49 ± 0.06
SRP ^(4)^	262	225 ± 96	78	339	-95.71 ± 45.65	0.57 ± 0.08
miRNA ^(4)^	1082	89 ± 16	55	153	-37.55 ± 8.89	0.46 ± 0.08
Guide ^(4)^	977	141 ± 47	66	459	-47.69 ± 23.52	0.51 ± 0.11
Total	6305	296 ± 445	29	2741	-106.93 ± 165.81	0.52 ± 0.09

^(1) ^Comparative RNA Web Site; ^(2) ^RNase P Database; ^(3) ^Genomic tRNA Database; ^(4) ^Rfam Database.

**Figure 1:**
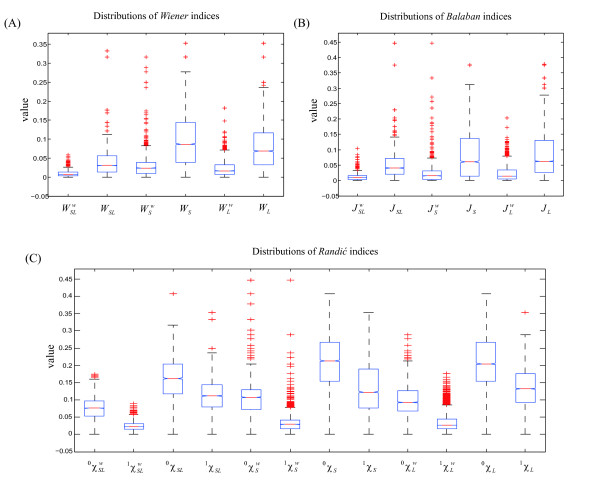
**Distributions of topological indices**. The distributions of the topological indices for the dataset of 6,305 ncRNAs are illustrated. (A) Distributions of *Wiener *indices. (B) Distributions of *Balaban *indices. (C) Distributions of *Randić *indices.

The distributions of our RNA topological indices based on the dataset are illustrated in Figure 1 [see Additional file 1]. Clearly, the statistical distributions of these indices cannot be well-described by Normal distribution model, since all of the distributions are skewed to some extent. There are four typical candidate distribution models that we considered for modeling the statistical distributions of our indices. These included the Normal and Log-normal distributions, the Gamma distribution, and the Weibull distribution. The parameters of these distribution models were estimated through the maximum likelihood method, and the goodness of model fit was evaluated by Pearson's correlation. The results of the distribution modeling listed in Table 2 revealed that the Weibull distribution (average goodness of fit was 0.94, 0.92 and 0.85 for *Wiener *indices, *Balaban *indices and *Randić *indices, respectively) and Gamma distribution (average goodness of fit were 0.93, 0.93 and 0.84 for *Wiener *indices, *Balaban *indices and *Randić *indices, respectively) fit the statistical distributions of these indices well, while the Log-normal distribution (average goodness of fit were 0.88, 0.89 and 0.77 for *Wiener *indices, *Balaban *indices and *Randić *indices, respectively) and the Normal distribution (average goodness of fitting are 0.84, 0.81 and 0.80 for *Wiener *indices, *Balaban *indices and *Randić *indices, respectively) failed to describe the distributions with sufficient accuracy. These results were verified with the distribution fitting results of the representatives of the three topological index families [see Additional file 2].

**Table 2:** Correlations between topological indices and their fitting models. Four typical distribution models (normal distribution, Gamma distribution, Weibull distribution and log-normal distribution) are employed here to model the statistical distributions of the three topological index families. The parameters of these distribution models are estimated through maximum likelihood method, and the goodness of model fitting is evaluated by Pearson's correlation coefficient. For each topological index, the highest Pearson's correlation coefficient is in bold.

Index family	Indices	Normal	Gamma	Weilbull	Log-normal
*Wiener*	$W_{SL}^{w}$	0.96	0.95	**0.97**	0.88
	*W*_*SL*_	0.83	**0.93**	**0.93**	0.83
	$W_{S}^{w}$	0.83	**1.00**	0.99	0.96
	*W*_*S*_	0.77	**0.93**	**0.93**	0.92
	$W_{L}^{w}$	0.93	0.94	**0.95**	0.85
	*W*_*L*_	0.74	**0.85**	**0.85**	0.81

*Balaban*	$J_{SL}^{w}$	0.91	0.99	**0.99**	0.95
	*J*_*SL*_	0.76	**0.91**	**0.91**	0.89
	$J_{S}^{w}$	0.82	**0.98**	0.96	0.93
	*J*_*S*_	0.81	**0.91**	0.89	0.89
	$J_{L}^{w}$	0.92	0.93	**0.94**	0.86
	*J*_*L*_	0.64	0.84	**0.84**	0.82

*Randić*	$\begin{array}{c} 0 \end{array}\chi_{SL}^{w}$	**0.93**	0.80	0.86	0.69
	$\begin{array}{c} 1 \end{array}\chi_{SL}^{w}$	0.84	**0.93**	**0.93**	0.88
	^0^*χ*_*SL*_	0.72	0.74	**0.75**	0.69
	^1^*χ*_*SL*_	**0.82**	0.79	0.81	0.71
	$\begin{array}{c} 0 \end{array}\chi_{S}^{w}$	0.95	**0.96**	**0.96**	0.89
	$\begin{array}{c} 1 \end{array}\chi_{S}^{w}$	0.82	**0.99**	0.98	0.94
	^0^*χ*_*S*_	0.57	0.57	**0.58**	0.51
	^1^*χ*_*S*_	0.87	**0.91**	**0.91**	0.86
	$\begin{array}{c} 0 \end{array}\chi_{L}^{w}$	0.92	0.96	**0.97**	0.87
	$\begin{array}{c} 1 \end{array}\chi_{L}^{w}$	0.80	**0.99**	**0.99**	0.95
	^0^*χ*_*L*_	0.52	0.53	**0.54**	0.51
	^1^*χ*_*L*_	0.81	0.85	**0.86**	0.80

Since all of the definitions of topological indices (equations (1) ~ (6) in Methods section) contained a summation operation, the topological indices examined herein may include information describing the shape and size of the secondary RNA molecule structure. The Pearson's correlations [see Additional file 3] showed that the *Wiener*-type and *Balaban*-type indices did not correlate strongly with the free energies and the lengths of the RNAs, as their values did not increase substantially with RNA size [see Additional file 4, 5, 7, 8]. However, most of the *Randić*-type indices did correlate strongly with the free energies and the lengths of the RNAs [see Additional file 6 and 9]. Furthermore, the topological indices appeared to be independent of GC contents [see Additional file 10, 11, 12]. Theses results are consistent with the conclusions drawn in computational chemistry [16].

#### Intercorrelations of topological indices

Clearly, no single topological index is sufficient to characterize the broad range of structure-function relationship studies on RNA molecule formation. Considering that various structural features of RNAs are usually correlated, the intercorrelations among topological indices should be examined when multiple topological indices are used. Moreover, it is useful to reduce the redundancy and create an orthogonal structural space.

**Figure 2:**
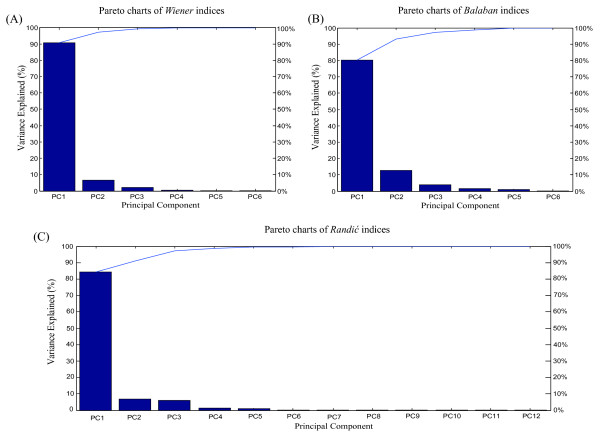
**Pareto charts of topological indices**. Pareto charts of three topological index families for the dataset of 6,305 ncRNAs are shown. The primary components in the Pareto chart are arranged in descending order. (A) Pareto charts of *Wiener *index family. (B) Pareto charts of *Balaban *index family. (C) Pareto charts of *Randić *index family.

We conducted correlation analysis and principal component analysis (PCA) on the RNA dataset listed in Table 1. These analyses reduce the complexity of the datasets and create new orthogonal variables from combinations of the original variables that describe spatial information. Figure 2 illustrates the Pareto charts of the three topological index families, whereby the primary principal components (PCs) are arranged in descending order, with the first PC, PC1, describing the greatest proportion of the variability being followed by PCs 2, 3, 4 and so on. In addition, the Pearson's correlations among the indices within the index families are presented [see Additional file 3, and 13, 14, 15]. These results indicate that *Wiener*-type and *Randić*-type indices are highly correlated within their families, and that the first three PCs of each index family contain more than 99% of the dataset variability, which comprises the information required to construct the indices. The correlation between the *Balaban*-type indices, however, appears to be weaker, as they require the first five PCs to explain 99% of the information.

### Application case studies

After defining the topological indices based on ECGs and analyzing their statistical properties, the questions naturally arose to regarding the potential utility of the knowledge of these indices. The answers came from the following three application case studies of our topological indices, in which they have been employed to quantify the structural aspects of RNA molecules.

#### Identification of miRNAs

Novel ncRNAs are difficult to detect experimentally, due to their short lengths, low expression levels, tissue specificity and lack of polyadenylation. Therefore, the most effective method for discovering ncRNAs may be computational identification of ncRNA candidates followed by biochemical verification [26]. Because of the strong inter-dependence between structure and function, incorporating structural features into ncRNA scanning programs could improve the accuracy of candidate identification. Based on secondary structure conservation, RNA structural information has been used in several ways in recently published works to identify microRNA (miRNA) candidates in select genomes [27-35]. The miRNAs molecules are abundant endogenous ~22-nucleotide (nt) noncoding RNAs that can play important roles in gene regulation at the post-transcriptional level. Roles include cleavage or translational repression through the binding of a minimal-recognition 'seed' sequence [36-39]. The miRNAs are transcribed as long primary molecules, which are processed into ~70 nt miRNA precursors (pre-miRNAs) that fold into a stem-loop hairpin structures via nuclear RNase III Drosha [12]. Mature miRNAs (~22 nt) are cleaved from pre-miRNAs through the action of Dicer endonuclease [40-42]. Throughout the miRNA biogenesis procedure, the hairpin structure of the pre-miRNA plays a crucial role, acting as the structure motif for expotin-5 in nuclear-cytoplasm transportation, and as a substrate for Dicer enzyme [13,41,43-46].

Although almost all pre-miRNAs are characterized by their stem-loop hairpin structures [28,29,35,47], a large number of pre-miRNA-like hairpins in many genomes can be folded. Distinguishing the real pre-miRNAs from other hairpin sequences with similar stem-loops (pseudo pre-miRNAs) is important both for understanding of the nature of miRNAs and for developing prediction methods for identifying miRNAs for which homology is unknown. However, this remains a challenging task. Xue *et al. *presented an SVM-based method for classifying real and pseudo pre-miRNAs [48]. A recent study distinguished real from pseudo pre-miRNAs using a random forest prediction model with a hybrid feature [49].

**Figure 3:**
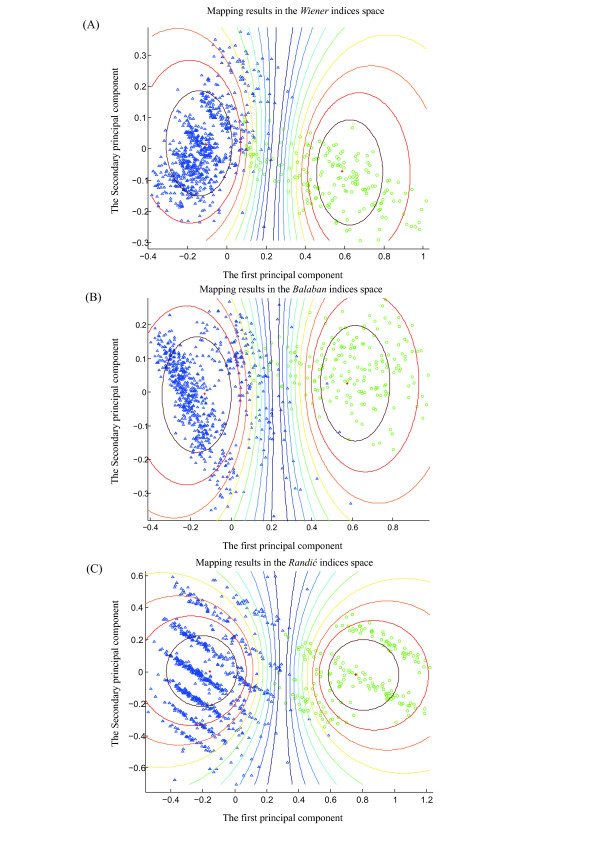
**Mapping results of miRNA identification**. The mapping results of miRNA identification using K-means clustering algorithm for the three topological index families are shown. In this application case study, 200 real pre-miRNAs are randomly chosen from the 1,082 miRNAs in dataset of Table 1, and the corresponding 1,000 pseudo pre-miRNAs are generated as reference set. Principal component analysis mapping method is employed here to visualize the clustering results for three types of topological indices. The green circle and blue upward-pointing triangle respectively represent real and pseudo pre-miRNAs, and the centroid is marked with red '+'. (A) Mapping result of the real and pseudo pre-miRNAs in the *Wiener *indices space. (B) Mapping result of real and pseudo pre-miRNAs in the *Balaba*n indices space. (C) Mapping result of real and pseudo pre-miRNAs in the *Randić *indices space.

**Figure 4:**
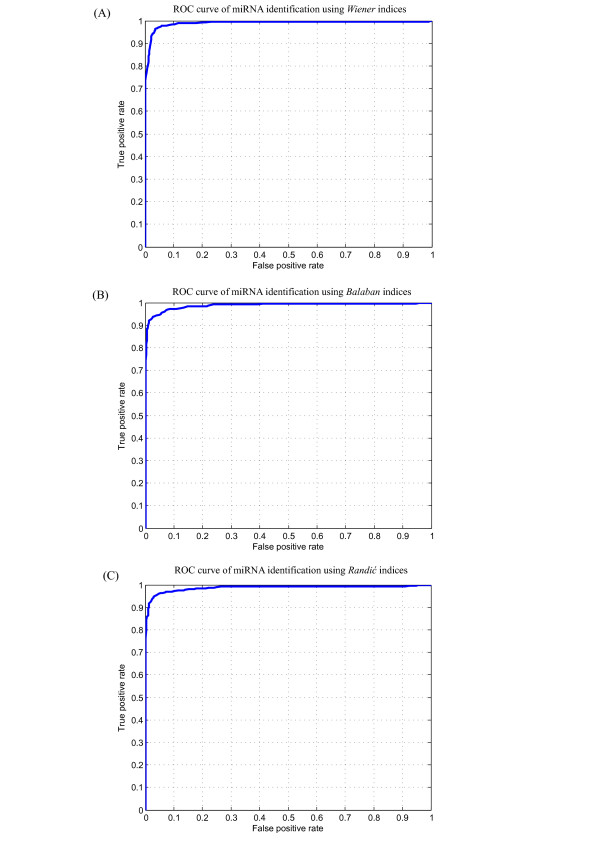
**ROC curves for miRNA identification**. ROC curves are employed here to evaluate and compare the performance of miRNA identification for three types of topological indices. (A) ROC curve for miRNA identification using *Wiener *indices. (B) ROC curve for miRNA identification using *Balaban *indices. (C) ROC curve for miRNA identification using *Randić *indices.

As numeric features of RNA structure, topological indices may be used to score candidates based on structure similarity measurements among the folds and structures of the reference miRNAs. We randomly chose 200 real pre-miRNAs from the 1,082 miRNAs in our dataset (Table 1) and generated 1,000 pseudo pre-miRNAs as a reference set using the dinucleotide shuffling method presented in our previous study [50]. To evaluate the potentials of topological indices as features in the miRNA identification procedure, we explored the distribution of the 200 real pre-miRNAs and the corresponding 1,000 pseudo pre-miRNAs in the topological feature space. Figures 3(A), (B) and 3(C) illustrate the 2D mapping results of these real and pseudo pre-miRNAs from the structural space to the topological feature space of the three types of topological indices using the K-means algorithm, respectively. The corresponding ROC curves are plotted in Figure 4.

We ran the K-means algorithm independently for 50 times, and each time randomly chose 200 real pre-miRNAs and generated corresponding 1,000 pseudo pre-miRNAs. The average accuracy of the miRNA identification was 0.968, 0.953 and 0.985 for *Wiener *indices, *Balaban *indices and *Randić *indices, respectively. The sensitivity and specificity exceeded 0.95 for all three types of topological indices. Table 3 shows the details of the evaluation results of the identifications performances, indicating that the performance of *Randić *indices was much higher than that of the *Wiener *indices and *Balaban *indices. This finding may be attributable to the high number of RNA structural details that are encoded into the 12 *Randić *indices.

**Table 3:** Evaluation results of miRNA identification. The evaluation results of miRNA identification using K-means clustering algorithm for the three topological index families are shown. In this application case study, the K-means algorithm is run independently for 50 times. For each test, 200 real pre-miRNAs are randomly chosen from the 1,082 miRNAs in dataset of Table 1 and the corresponding 1,000 pseudo pre-miRNAs are generated as reference set. The clustering accuracy, sensitivity and specificity are employed here to evaluate the performance of the identification results for *Wiener *indices, *Balaban *indices and *Randić *indices, respectively.

Index	Clustering accuracy			Sensitivity			Specificity		
	
	Mean ± SD	Min	Max	Mean ± SD	Min	Max	Mean ± SD	Min	Max
*Balaban*	0.9534 ± 0.005	0.9475	0.9658	0.9597 ± 0.0072	0.955	0.980	0.9621 ± 0.0068	0.953	0.968
*Wiener*	0.968 ± 0.0014	0.9658	0.9708	0.9623 ± 0.0026	0.957	0.985	0.9891 ± 0.002	0.986	0.993
*Randić*	0.9849 ± 0.0026	0.9808	0.9908	0.9842 ± 0.0047	0.975	0.990	0.9851 ± 0.0033	0.979	0.992

#### Classification of ncRNAs

With the rapidly increasing knowledge of the cellular roles of RNA molecules [51,52], the expanding repertoire of known functional RNAs has spurred renewed efforts to catalogue and classify RNA structures. An understanding of structural diversity in RNA populations is crucial for identifying novel RNA structures and pursuing RNA genomics initiatives. Since RNA secondary topologies are remarkably well conserved across functional classes, their topological characteristics provide a basis for organizing RNA secondary structures on a broad scale [53]. In this report, we used topological indices to catalogue and to classify RNA structures based on the correlations between conserved RNA secondary structures and topological indices. This method is similar to that of RNA-As-Graphs (RAG) [8,21], which classifies RNA structures based on the topological properties of their secondary motifs using graph theory results.

**Figure 5:**
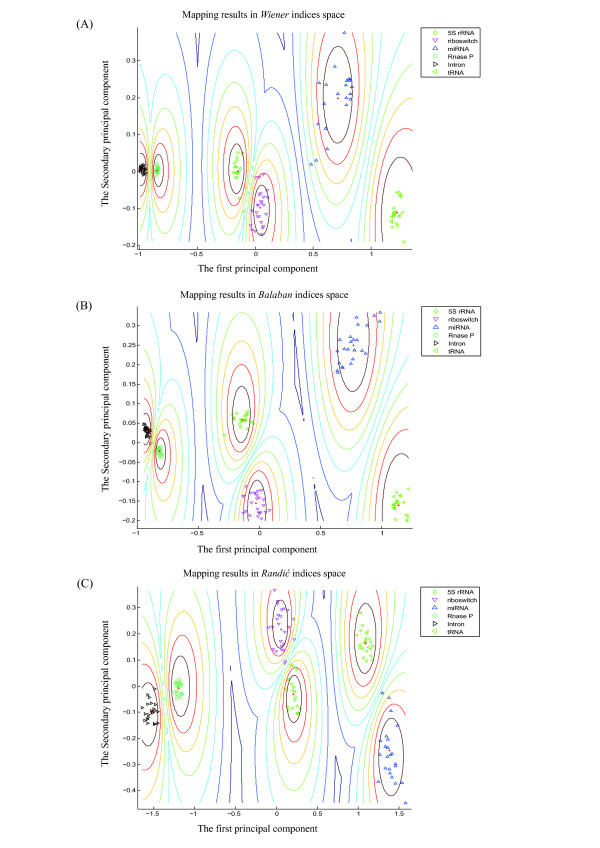
**Mapping results of ncRNA classification**. The mapping results of ncRNA classification using K-means clustering algorithm for the three topological index families are shown. In this application case study, 25 sequences of each kind are randomly chosen from six kinds of ncRNAs (5S rRNA, riboswitch, miRNA, RNase P, Intron, and tRNA) listed in Table 1. Principal component analysis mapping method is employed here to visualize the clustering results for the three topological index families. The centroid of each kind of ncRNAs is marked with red '+'. (A) Mapping results of six kinds of ncRNAs in the *Wiener *indices space. (B) Mapping results of six kinds of ncRNAs in the *Balaban *indices space. (C) Mapping results of six kinds of ncRNAs in the *Randić *indices space.

We randomly chose 25 sequences from each of the six RNA classes (5S rRNA, riboswitch, miRNA, RNase P, Intron, and tRNA; Table 1). The 2D mapping results of the K-means classification is shown in Figure 5, with the ncRNA centroids demarcated. We ran the K-means algorithm independently 50 times, and randomly chose 25 sequences from each class each time. The average clustering accuracy was about 98.0% for the three types of topological indices.

#### Deleterious mutation analysis of RNA

Mutations in RNA genes may lead to striking alterations in the 2D RNA structures that impair cellular functions, resulting in certain diseases [54]. For example, mutations of tRNAs in mitochondria were reported to harbor more than half of the known mitochondrial pathogenic mutations [55]. Recent research has further shown that mutations in miRNA genes and their flanking sequences may contribute to cancer [56-58]. On the other hand, deleterious RNA mutations in pathogenic species can be exploited. Yassin *et al *demonstrated that deleterious mutations in bacterial rRNAs can serve as hallmarks of antibiotic sites [59]. Additionally, in their study on influenza viruses, Herlocher *et al*. found a nonsense mutation on a PB2 segment that caused monumental differences in the RNA secondary structure; a finding that can be used to make a live vaccine [60].

In principle, an RNA mutation can be deleterious when it disrupts a functional site involved in catalysis, ligand-binding or protein interactions. Since ncRNA function depends critically on its secondary structure, nucleotide alterations that result in structural changes have great potential to be deleterious. Accordingly, structure analysis should help to identify deleterious mutations. Some structure-based methods and software for RNA deleterious mutation analysis have been reported [17,18,24,61,62].

**Figure 6:**
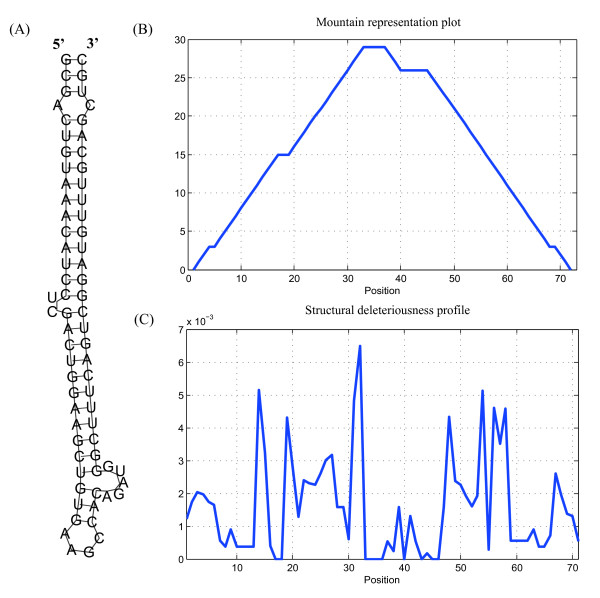
**Deleterious mutation analysis of miRNA**. The results of deleterious mutation analysis for miRNA miR-30a precursor are shown. (A) The secondary structure of wild-type miR-30a. (B) The mountain representation plot of the structure of wild-type miR-30a. (C) Structural deleteriousness profile of miR-30a estimated by weighted first order *Randić *index.

To test how our topological indices can help with deleterious RNA mutation analysis, we analyzed the precursor of human miRNA miR-30a (pre-miR-30a), a stem-loop of 71 nt (Figure 6A). Figure 6B shows its mountain representation [63]. The dissimilarity of the secondary structures between the wild-type RNAs and those with possible single point mutations are measured by computing the differences between the weighted first order *Randić *indices. The mean structural differences among the wild-type and the possible mutants at each position were extracted into a structural deleteriousness profile [62] and plotted as waveforms (Figure 6C); the sites that were crucial for structure determination are represented by peaks with high structural deleteriousness within the profile.

It appeared that the mutations opening the base stem of the precursor led to marked differences in RNA structure, while the mutations in the terminal loop and bulges seemed to be less deleterious. This finding indicates that the base-pairing at the base of the precursor stem is of critical importance to RNA structure determination compared to the internal loops, terminal loops and bulges. These results are in good accord with the same conclusions drawn in previous experimental studies [12,13,15].

## Methods

### Element-contact graph representations for RNA secondary structure

**Figure 7:**
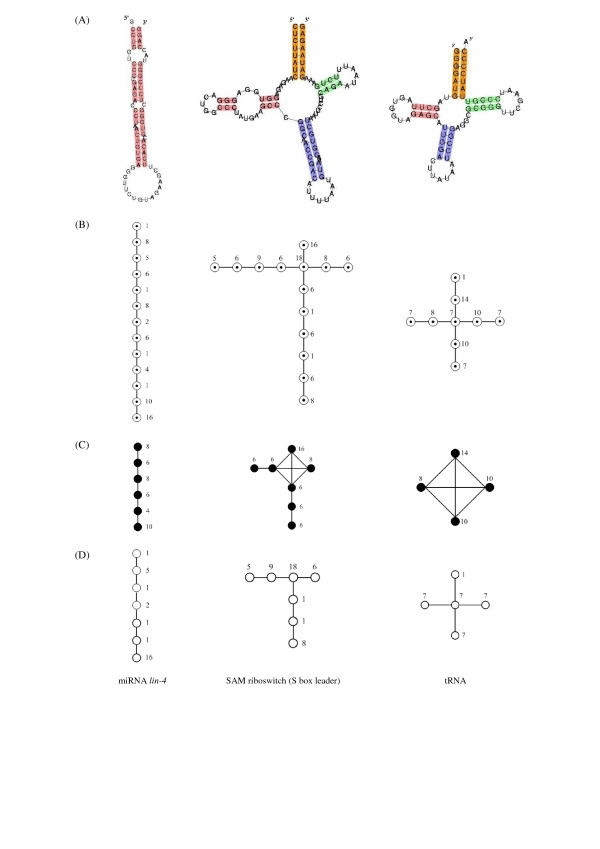
**Element-contact graph representations for three typical RNA secondary structures**. Three typical RNA secondary structures and their element-contact graph representations are illustrated. (A) Secondary structures of three typical RNAs (miRNA *lin-4*, SAM riboswitch, tRNA). (B) Stem-loop-contact graphs of the three typical RNAs. (C) Stem-contact graphs of the three typical RNAs. (D) Loop-contact graphs of the three typical RNAs.

To establish a comprehensive basis for new RNA structure descriptors, and to avoid the use of colored graphics, we used three distinct non-colored ECGs compensated by one another to characterize the secondary structure of an RNA molecule. Similar to the classical HIT, the topology of all structural elements in RNA secondary structure are represented in a stem-loop-contact graph (SLCG), in which the stems and loops are all assigned as vertices (□) without differences, and the edges (-) represent connection relationships. Two other ECGs derived from SLCG are stem-contact graph (SCG) and loop-contact graph (LCG), describing stem and loop topology, respectively. The relationship between the usual form of typical RNA secondary structures and their element-contact graph representations are illustrated in Figure 7. In a LCG, as with some classical RNA graphs [5,6,9], stem elements are abstracted into the edges (-) between loop elements, while loop elements are represented as vertices (○). In a SCG, however, the stem topology cannot be obtained by simply abstracting the loops into vertices (●), and stems into edges (-) conversely, since the branches of the RNA graph always end with loop elements. Only the loops between two or more stems can be described as edges; hairpins and external loops cannot be described in the SCG. Stems connected with multiple loops are considered to be adjacent to each other and therefore joined with edges. The stem elements in the SLCG and SCG, distinct from the HIT, are all weighted by the number of nucleotides included.

Formally, these three types of ECGs can be represented as ordered triples of disjoint sets *G*_*SL *_= (*V*_*SL*_, *E*_*SL*_, *W*_*SL*_), GS = (*V*_*S*_, *E*_*S*_, *W*_*S*_) and *G*_*L *_= (*V*_*L*_, *E*_*L*_, *W*_*L*_), respectively, where *V*_*SL*_, *V*_*S*_, *V*_*L *_are a set of vertices, *E*_*SL*_, *E*_*S*_, *E*_*L *_are a set of edges, and *W*_*SL*_, *W*_*S*_, *W*_*L *_are a set of weights. The group of these three ECGs *G*_*E *_= {*G*_*SL*_, *G*_*S*_, *G*_*L*_} forms a complete and superlative description of RNA secondary structure, which facilitates the definition of topological indices. Although there are some redundancies, all of these ECGs contribute importantly to the final analysis.

### Classical topological indices based on ECGs

Most topological indices used in computational chemistry can be extended easily into ECGs to characterize RNA secondary structure. In our study, three of the most widely used topological indices were redefined in ECGs for application testing, comprised of the *Wiener*, *Randić *and *Balaban *indices. These indices are essentially the mathematical properties of a graph characterizing its 'compactness'.

As the first non-trivial topological index, the *Wiener *index has become one of the most widely utilized and investigated topological indices, as it is simple to compute and offers good structure-property correlations in QSAR and QSPR studies. The *Wiener *index of a graph *G *is the half-sum of all entries in the distance matrix **D **= [*d*_*ij*_], i.e.

(1) $$W(G)=\sum_{i<j}{\left(d_{ij}\right)}^{\alpha }$$

*Wiener*-type indices can be defined for all molecular graph matrices with the *Wiener *operator. Suggested by Merris [64,65] and tested by Barash's group [17,18], the *Wiener *index has been introduced into a fine-grained RNA graph, in which each nucleotide becomes a node of the graph [66,67]. Thus, the classic *Wiener *indices increase rapidly with the magnitude of a graph, especially for the weighted *Wiener *indices. This may be the main reason why Avihoo and Barash limit their *Wiener *index to fine-grained RNA graphs that characterize only small RNAs (≤ 50 nt) [67]. In this study, similar to the work on the connectivity index [68], we generalized the *Wiener *indices by assigning *α *= -0.5 to the exponent of each item in the equation (1) to reduce their range.

The *Balaban *index of a graph *G *also is a distance-based graph connectivity index, defined as

(2) $$J(G)=\frac{q}{\mu +1}\sum_{v_{i}v_{j}}{\left(D_{i}D_{j}\right)}^{\beta }$$

where *D*_*i *_and *D*_*j *_denote the distance sums of the vertices *v*_*i *_and *v*_*j*_, and can be easily computed by summarizing corresponding rows or columns in the distance matrix, *q *is the number of edges in the molecular graph, *μ *is the cyclomatic number and the summation goes over all edges in the graph.

The *Randić *indices of ECGs encode aspects of element connectivity for RNA secondary structure. The *m *th order *Randić *index of a graph *G *is given as

(3) χm(G)=∑vi1vi2⋯vim+1(δi1δi2⋯δim+1)γ

where *δ*_*v *_is the degree of vertex *v *and the summation is over the total number of sub-graphs of order *m*. The first two order *Randić *indices, ^0^*χ *and ^1^*χ*, are employed in this study.

As vertex-weighted RNA graphs, ECGs offer convenience for constructing weighted numerical descriptors aimed at detailed structure characterization. The method presented by Zmazek and Zrovnik [69] is employed for extending the indices mentioned above, and the properties of vertices in equation (1) ~ (3) are multiplied by their weights. The weighted *Wiener *index, and the *Balaban *index and *Randić *indices of a graph are given as:

(4) $$W(G)=\sum_{i<j}{\left(w_{i}w_{j}d_{ij}\right)}^{\alpha }$$

(5) $$J(G)=\frac{q}{\mu +1}\sum_{v_{i}v_{j}}{\left(w_{i}D_{i}\cdot w_{j}D_{j}\right)}^{\beta }$$

(6) χm(G)=∑vi1vi2⋯vim+1(wi1δi1⋅wi2δi2⋅⋯⋅wim+1δim+1)γ

where *w*_*i *_and *w*_*j *_are the weights of vertex *v*_*i *_and *v*_*j*_, respectively.

Both the weighted and the unweighted topological indices are examined in this study to evaluate their utility and potential in structure determination applications. The exponents of each item in equations (1) ~ (6) are assigned to *α *= -0.5, *β *= -0.5, and *γ *= -0.5 to reduce their ranges, respectively. The symbols representing these indices are listed in Table 4.

**Table 4:** The symbols of the three topological index families. The symbols of the three topological index families based on element-contact graphs are shown.

Index family	Indices based on SLCG		Indices based on SCG		Indices based on LCG	
	
	weighted	unweighted	weighted	unweighted	weighted	unweighted
*Wiener*	$W_{SL}^{w}$	*W*_*SL*_	$W_{S}^{w}$	*W*_*S*_	$W_{L}^{w}$	*W*_*L*_
*Balaban*	$J_{SL}^{w}$	*J*_*SL*_	$J_{S}^{w}$	*J*_*S*_	$J_{L}^{w}$	*J*_*L*_
*Randić*	$\begin{array}{c} 0 \end{array}\chi_{SL}^{w}$, $\begin{array}{c} 1 \end{array}\chi_{SL}^{w}$	^0^*χ*_*SL*_, ^1^*χ*_*SL*_	$\begin{array}{c} 0 \end{array}\chi_{S}^{w}$, $\begin{array}{c} 1 \end{array}\chi_{S}^{w}$	^0^*χ*_*S*_, ^1^*χ*_*S*_	$\begin{array}{c} 0 \end{array}\chi_{L}^{w}$, $\begin{array}{c} 1 \end{array}\chi_{L}^{w}$	^0^*χ*_*L*_, ^1^*χ*_*L*_

### Dataset of ncRNAs

To explore the relationship among the topological indices and RNA secondary structures, we have selected a dataset of 6,305 ncRNAs as representatives of the human RNA population and have evaluated their topological indices. We divided these 6,305 ncRNAs into two classes. One class covers five ncRNA types with known structures, obtained from the Comparative RNA Web Site [70], RNase P database [71] and the Genomic tRNA Database [72]. The second class is composed of six ncRNA types with secondary structures predicted by the Vienna RNA package [73]. All of these ncRNAs were obtained from Rfam [53]. Table 1 provides a detailed description of the dataset.

### Clustering algorithm, and its performance evaluation and visualization

The K-means algorithm [74] is one of the most important and most widespread approaches to prototype-based clustering. The K-means methodology is based on the idea that a center point can represent a cluster. Thus, K-means defines a prototype in terms of a centroid, which is usually the mean or median point of a group of points. Herein, we used the PCA mapping method to visualize the 'RNA spaces' of the clustering results, which is very useful in the analysis and visualization of the correlated high-dimensional data.

We used the clustering accuracy as a measure of a clustering result. Given the final number of clusters, *K*, clustering accuracy *r *is defined as

(7) $$r=\frac{\sum_{i=1}^{K}r_{i}}{n}$$

where *n *is the number of instances in the data set and *r*_*i *_is the number of instances partitioned into the correct cluster *i*. For miRNA identification, we use receiver operating characteristic (ROC) curves to evaluate and compare the classification performance. The ROC curve provides a convenient graphical display of the trade-off between true- and false-positive rates. Additional terms associated with ROC curves are sensitivity and specificity [75].

## Discussion and Conclusion

This paper presents a complete and fine-grained topological description for representing RNA graphs, and establishes a new basis for constructing RNA topological indices. Distinct from other methods, RNA secondary structure is represented by a combination of three vertex-weighted element-contact graphs. Based on the opinion that the stem and loop regions in RNA molecules have similar importance in biochemical processes, the stem and loop topologies are described in stem-contact and loop-contact graphs, respectively, while the overall pattern of the structure is abstracted into a stem-loop-contact graph. In addition, these graphs can be selected according to the needs of a particular application. Three typical topological index families defined with ECGs are described.

To investigate the relationship between the topological indices and RNA secondary structures, we constructed a detailed analysis on a dataset of 6,305 ncRNA sequences downloaded from different databases, and explored the numerical features of these indices. We then employed the topological indices to quantify the structural aspects of the selected RNAs, and utilized them to identify miRNAs, classify ncRNAs and conduct deleterious mutation analyses. Based on the applied small dataset, we find that the topological indices can distinguish true from pseudo pre-miRNAs with about 96% accuracy, and cluster known types of ncRNAs with about 98% accuracy. The results indicate that the topological indices can characterize RNA structure details, and show high potential for identifying and classifying ncRNAs. Importantly, while difficult, the successful identification and classification of ncRNAs may provide a new approach for discovering new ncRNAs. The difficulty of correctly identifying and classifying these molecules is underscored by the fact that the predictions of both Evofold [76] and RNAz [77] differ to some extent from that of the ENCODE [78] experimental data. Further research is needed to fully resolve the challenging problem of predicting and classifying ncRNAs.

The utility test and the application examples of typical topological indices defined on the ECGs illustrate their latent utility for RNA structure analysis. With the aid of topological indices, it is now possible for biologists to explore 'RNA spaces' visually, as exemplified by the three case studies presented herein. Characterizing RNA molecules using topological indices may open a door to studying the structure-function relationships of RNA molecules by combining many application algorithms for pattern recognition and classification, most of which are based on feature space. Further applications of these topological indices are represented by our studies on robustness analysis of RNA secondary structure [50,79], whereby the topological indices are employed as distance measures for secondary structures to evaluate the robustness of RNAs.

## Competing interests

The author(s) declare that they have no competing interests.

## Authors' contributions

WS wrote the programs, analyzed the results and drafted the manuscript. XB and ZZ helped in analysis and discussion, and gave useful comments. SW and XB guided the project. All authors read and approved the final manuscript.

## Supplementary Material

Additional file 1 Supplementary table S1 – S3. Calculation of *Wiener*, *Balaban*, *Randić *indices based on the dataset of 6,305 ncRNAs. Click here for file

Additional file 2 Distribution Fitting results for three representatives of topological indices. The distribution fitting results for three representatives of topological indices based on the dataset of 6,305 ncRNAs are shown. Four typical distribution models (normal distribution, Gamma distribution, Weibull distribution and log-normal distribution) are employed here to model the statistical distributions of the three topological index families. (A) Probability distribution curves (left) and Cumulative distribution curves (right) of weighted *Weiner *index based on SLCG representation. (B) Probability distribution curves (left) and Cumulative distribution curves (right) of weighted *Balaban *index based on SLCG representation. (C) Probability distribution curves (left) and Cumulative distribution curves (right) of weighted zero order *Randić *index based on SLCG representation. Click here for file

Additional file 3 Supplementary table S4 – S7. Statistical properties of topological indices. Click here for file

Additional file 4 Correlations between *Wiener *indices and the free energy of RNA. Correlations between *Wiener *indices and free energy for the dataset of 6,305 ncRNAs are shown. For convenience of visualization, both X and Y axes are scaled logarithmically. Click here for file

Additional file 5 Correlations between *Balaban *indices and the free energy of RNA. Correlations between *Balaban *indices and free energy for the dataset of 6,305 ncRNAs are shown. For convenience of visualization, both X and Y axes are scaled logarithmically. Click here for file

Additional file 6 Correlations between *Randić *indices and the free energy of RNA. Correlations between *Randić *indices and free energy for the dataset of 6,305 ncRNAs are shown. For convenience of visualization, both X and Y axes are scaled logarithmically. Click here for file

Additional file 7 Correlations between *Wiener *indices and the length of RNA. Correlations between *Wiener *indices and length for the dataset of 6,305 ncRNAs are shown. For convenience of visualization, both X and Y axes are scaled logarithmically. Click here for file

Additional file 8 Correlations between *Balaban *indices and the length of RNA. Correlations between *Balaban *indices and length for the dataset of 6,305 ncRNAs are shown. For convenience of visualization, both X and Y axes are scaled logarithmically. Click here for file

Additional file 9 Correlations between *Randić *indices and the length of RNA. Correlations between *Randić *indices and length for the dataset of 6,305 ncRNAs are shown. For convenience of visualization, both X and Y axes are scaled logarithmically. Click here for file

Additional file 10 Correlations between *Wiener *indices and the GC content of RNA. Correlations between *Wiener *indices and GC content for the dataset of 6,305 ncRNAs are shown. For convenience of visualization, both X and Y axes are scaled logarithmically. Click here for file

Additional file 11 Correlations between *Balaban *indices and the GC content of RNA. Correlations between *Balaban *indices and GC content for the dataset of 6,305 ncRNAs are shown. For convenience of visualization, both X and Y axes are scaled logarithmically. Click here for file

Additional file 12 Correlations between *Randić *indices and the GC content of RNA. Correlations between *Randić *indices and GC content for the dataset of 6,305 ncRNAs are shown. For convenience of visualization, both X and Y axes are scaled logarithmically. Click here for file

Additional file 13 Correlations between *Wiener *indices. Correlations between *Wiener *indices for the dataset of 6,305 ncRNAs are shown. The diagonal figures show the distributions of the *Wiener *indices. Click here for file

Additional file 14 Correlations between *Balaban *indices. Correlations between *Balaban *indices for the dataset of 6,305 ncRNAs are shown. The diagonal figures show the distributions of the *Balaban *indices. Click here for file

Additional file 15 Correlations between *Randić *indices. Correlations between *Randić *indices for the dataset of 6,305 ncRNAs are shown. The diagonal figures show the distributions of the *Randić *indices. Click here for file
