# Supplementary material for: A novel representation of RNA secondary structure based on element-contact graphs
Source: BMC Bioinformatics. 2008 Apr 11;9:188. doi: 10.1186/1471-2105-9-188 (PMC2373570; doi:10.1186/1471-2105-9-188)
Supplement: Additional file 2 — Distribution Fitting results for three representatives of topological indices. The distribution fitting results for three representatives of topological indices based on the dataset of 6,305 ncRNAs are shown. Four typical distribution models (normal distribution, Gamma distribution, Weibull distribution and log-normal distribution) are employed here to model the statistical distributions of the three topological index families. (A) Probability distribution curves (left) and Cumulative distribution curves (right) of weighted Weiner index based on SLCG representation. (B) Probability distribution curves (left) and Cumulative distribution curves (right) of weighted Balaban index based on SLCG representation. (C) Probability distribution curves (left) and Cumulative distribution curves (right) of weighted zero order Randić index based on SLCG representation. [file 1471-2105-9-188-S2.pdf]

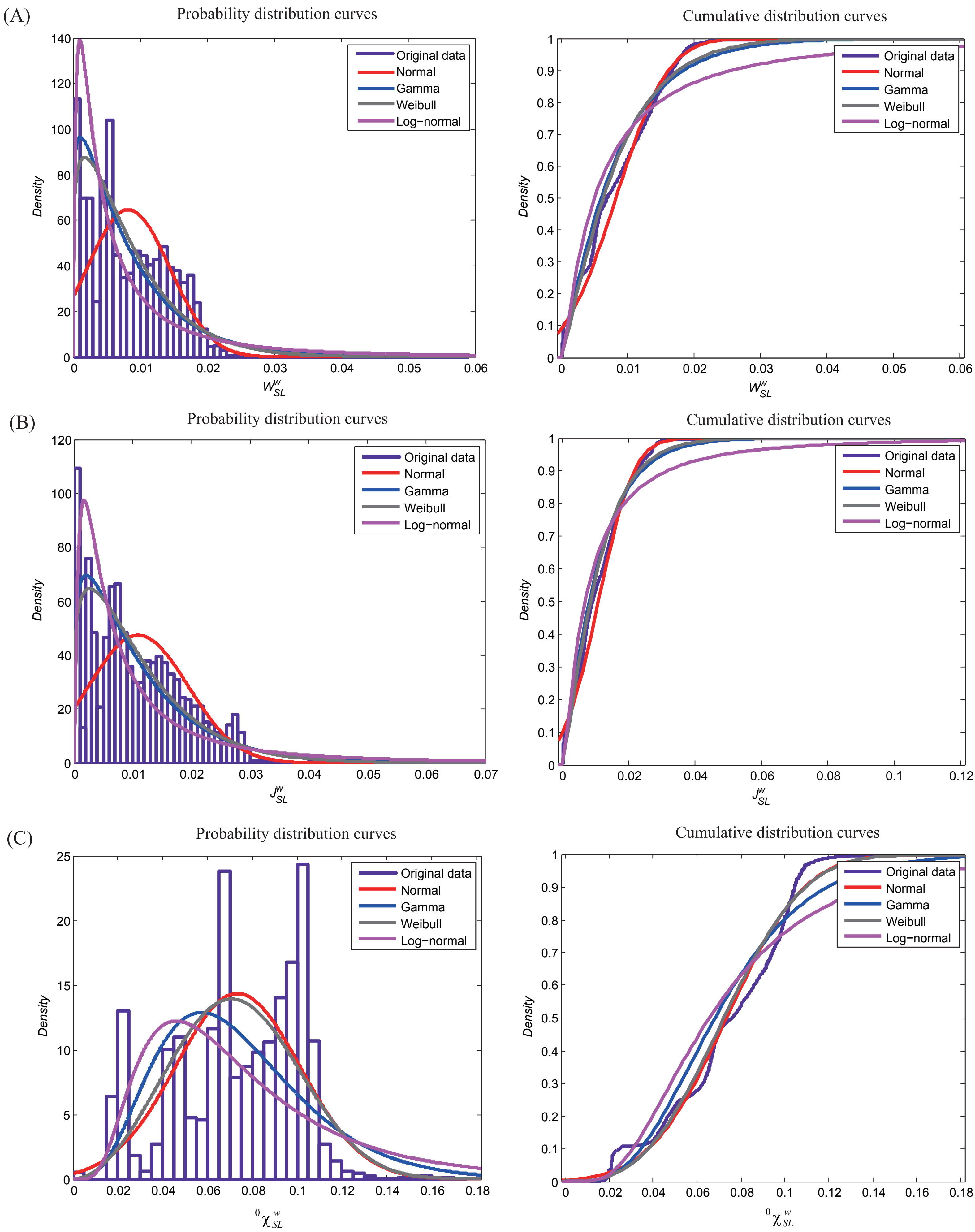
(A)
Probability distribution curves
Cumulative distribution curves
Density
W^{w}_{SL}
Original data
Normal
Gamma
Weibull
Log−normal
(B)
Probability distribution curves
Cumulative distribution curves
Density
J^{w}_{SL}
Original data
Normal
Gamma
Weibull
Log−normal
(C)
Probability distribution curves
Cumulative distribution curves
Density
${}^{0}\chi^{w}_{SL}$
Original data
Normal
Gamma
Weibull
Log−normal
