# Supplementary material for: A novel representation of RNA secondary structure based on element-contact graphs
Source: BMC Bioinformatics. 2008 Apr 11;9:188. doi: 10.1186/1471-2105-9-188 (PMC2373570; doi:10.1186/1471-2105-9-188)
Supplement: Additional file 3 — Supplementary table S4 – S7. Statistical properties of topological indices. [file 1471-2105-9-188-S3.doc]

## Supplementary Tables S4 – S7

**Supplementary Table S4. Correlations between topological indices and free energy, the length and GC content of RNA.**

Correlation analysis between the three topological index families and the free energy, the length and GC content of ncRNAs on the dataset of 6,305 ncRNAs is made. The Pearson’s correlation coefficients are presented for *Wiener*-type indices, *Balaban*-type indices, and *Randić*-type indices.

| Index family | Indices | dG | Length | GC% |
| --- | --- | --- | --- | --- |
| *Wiener* | $W_{SL}^{w}$ | 0.55 | -0.57 | 0.08 |
|  | $W_{SL}$ | 0.48 | -0.49 | 0.03 |
|  | $W_{S}^{w}$ | 0.43 | -0.43 | 0.06 |
|  | $W_{S}$ | 0.50 | -0.51 | 0.04 |
|  | $W_{L}^{w}$ | 0.50 | -0.53 | 0.03 |
|  | $W_{L}$ | 0.54 | -0.55 | 0.04 |
| *Balaban* | $J_{SL}^{w}$ | 0.52 | -0.54 | 0.08 |
|  | $J_{SL}$ | 0.47 | -0.48 | 0.06 |
|  | $J_{S}^{w}$ | 0.35 | -0.36 | -0.03 |
|  | $J_{S}$ | 0.44 | -0.45 | -0.08 |
|  | $J_{L}^{w}$ | 0.42 | -0.44 | 0.08 |
|  | $J_{L}$ | 0.42 | -0.44 | 0.14 |
| *Randić* | ${}^{0}\chi_{SL}^{w}$ | 0.73 | -0.76 | 0.07 |
|  | ${}^{1}\chi_{SL}^{w}$ | 0.59 | -0.62 | 0.02 |
|  | ${}^{0}\chi_{SL}$ | 0.72 | -0.74 | 0.03 |
|  | ${}^{1}\chi_{SL}$ | 0.70 | -0.72 | 0.00 |
|  | ${}^{0}\chi_{S}^{w}$ | 0.71 | -0.72 | 0.02 |
|  | ${}^{1}\chi_{S}^{w}$ | 0.42 | -0.43 | -0.03 |
|  | ${}^{0}\chi_{S}$ | 0.72 | -0.73 | -0.03 |
|  | ${}^{1}\chi_{S}$ | 0.55 | -0.57 | -0.11 |
|  | ${}^{0}\chi_{L}^{w}$ | 0.64 | -0.67 | 0.03 |
|  | ${}^{1}\chi_{L}^{w}$ | 0.44 | -0.47 | -0.03 |
|  | ${}^{0}\chi_{L}$ | 0.72 | -0.74 | 0.05 |
|  | ${}^{1}\chi_{L}$ | 0.64 | -0.66 | 0.02 |

**Supplementary Table S5. Intercorrelations between *Wiener* indices.**

Intercorrelation analysis between *Wiener* indices on the dataset of 6,305 ncRNAs is made, and the Pearson’s correlation coefficients are presented.

| Index | $W_{SL}^{w}$ | $W_{SL}$ | $W_{S}^{w}$ | $W_{S}$ | $W_{L}^{w}$ | $W_{L}$ |
| --- | --- | --- | --- | --- | --- | --- |
| $W_{SL}^{w}$ | 1.00 |  |  |  |  |  |
| $W_{SL}$ | 0.94 | 1.00 |  |  |  |  |
| $W_{S}^{w}$ | 0.85 | 0.88 | 1.00 |  |  |  |
| $W_{S}$ | 0.93 | 0.99 | 0.91 | 1.00 |  |  |
| $W_{L}^{w}$ | 0.93 | 0.85 | 0.66 | 0.80 | 1.00 |  |
| $W_{L}$ | 0.95 | 0.98 | 0.80 | 0.96 | 0.89 | 1.00 |

**Supplementary Table S6. Intercorrelations between *Balaban* indices.**

Intercorrelation analysis between *Balaban* indices is made, and the Pearson’s correlation coefficients are presented.

| Index | $J_{SL}^{w}$ | $J_{SL}$ | $J_{S}^{w}$ | $J_{S}$ | $J_{L}^{w}$ | $J_{L}$ |
| --- | --- | --- | --- | --- | --- | --- |
| $J_{SL}^{w}$ | 1.00 |  |  |  |  |  |
| $J_{SL}$ | 0.94 | 1.00 |  |  |  |  |
| $J_{S}^{w}$ | 0.76 | 0.69 | 1.00 |  |  |  |
| $J_{S}$ | 0.85 | 0.82 | 0.88 | 1.00 |  |  |
| $J_{L}^{w}$ | 0.83 | 0.75 | 0.49 | 0.68 | 1.00 |  |
| $J_{L}$ | 0.85 | 0.91 | 0.47 | 0.65 | 0.85 | 1.00 |

**Supplementary Table S7. Intercorrelations between *Randić* indices.**

Intercorrelation analysis between *Randić* indices is made, and the Pearson’s correlation coefficients are presented.

| Index | ${}^{0}\chi_{SL}^{w}$ | ${}^{1}\chi_{SL}^{w}$ | ${}^{0}\chi_{SL}$ | ${}^{1}\chi_{SL}$ | ${}^{0}\chi_{S}^{w}$ | ${}^{1}\chi_{S}^{w}$ | ${}^{0}\chi_{S}$ | ${}^{1}\chi_{S}$ | ${}^{0}\chi_{L}^{w}$ | ${}^{1}\chi_{L}^{w}$ | ${}^{0}\chi_{L}$ | ${}^{1}\chi_{L}$ |
| --- | --- | --- | --- | --- | --- | --- | --- | --- | --- | --- | --- | --- |
| ${}^{0}\chi_{SL}^{w}$ | 1.00 |  |  |  |  |  |  |  |  |  |  |  |
| ${}^{1}\chi_{SL}^{w}$ | 0.92 | 1.00 |  |  |  |  |  |  |  |  |  |  |
| ${}^{0}\chi_{SL}$ | 0.94 | 0.76 | 1.00 |  |  |  |  |  |  |  |  |  |
| ${}^{1}\chi_{SL}$ | 0.95 | 0.81 | 0.99 | 1.00 |  |  |  |  |  |  |  |  |
| ${}^{0}\chi_{S}^{w}$ | 0.93 | 0.84 | 0.89 | 0.92 | 1.00 |  |  |  |  |  |  |  |
| ${}^{1}\chi_{S}^{w}$ | 0.66 | 0.7 | 0.59 | 0.66 | 0.84 | 1.00 |  |  |  |  |  |  |
| ${}^{0}\chi_{S}$ | 0.95 | 0.83 | 0.96 | 0.98 | 0.95 | 0.72 | 1.00 |  |  |  |  |  |
| ${}^{1}\chi_{S}$ | 0.84 | 0.84 | 0.81 | 0.88 | 0.90 | 0.84 | 0.93 | 1.00 |  |  |  |  |
| ${}^{0}\chi_{L}^{w}$ | 0.94 | 0.94 | 0.84 | 0.87 | 0.79 | 0.55 | 0.86 | 0.8 | 1.00 |  |  |  |
| ${}^{1}\chi_{L}^{w}$ | 0.78 | 0.9 | 0.63 | 0.69 | 0.62 | 0.48 | 0.68 | 0.72 | 0.93 | 1.00 |  |  |
| ${}^{0}\chi_{L}$ | 0.94 | 0.76 | 0.99 | 0.97 | 0.85 | 0.53 | 0.93 | 0.77 | 0.87 | 0.67 | 1.00 |  |
| ${}^{1}\chi_{L}$ | 0.90 | 0.79 | 0.93 | 0.95 | 0.8 | 0.54 | 0.89 | 0.79 | 0.89 | 0.76 | 0.97 | 1.00 |
