# Supplementary figures and images for: A novel representation of RNA secondary structure based on element-contact graphs
Source: BMC Bioinformatics. 2008 Apr 11;9:188. doi: 10.1186/1471-2105-9-188 (PMC2373570; doi:10.1186/1471-2105-9-188)

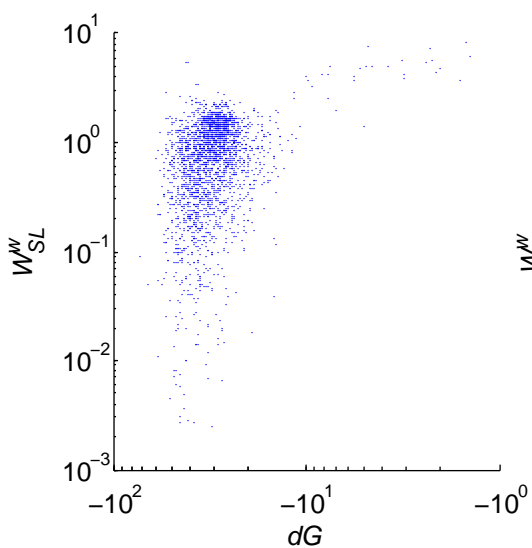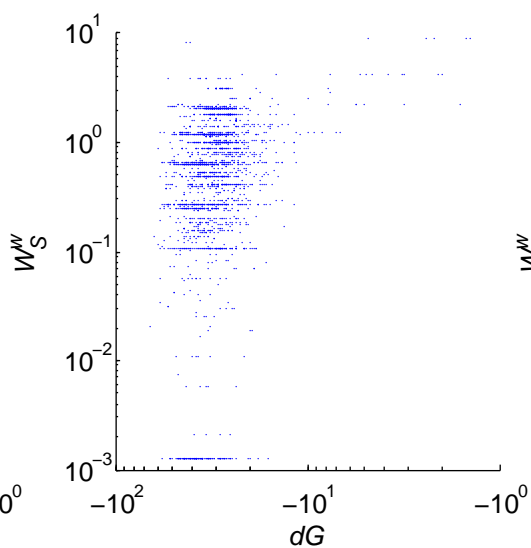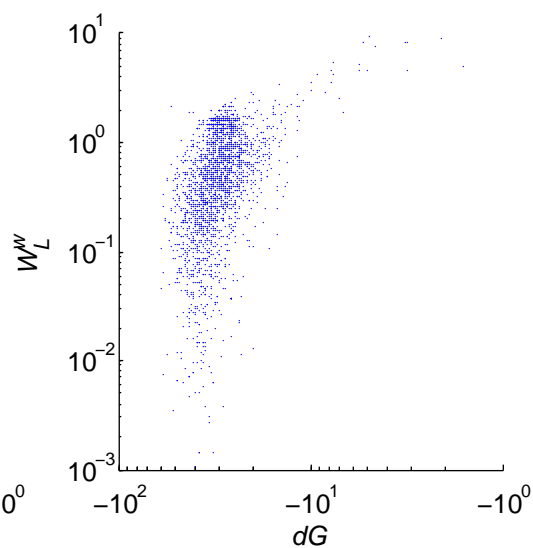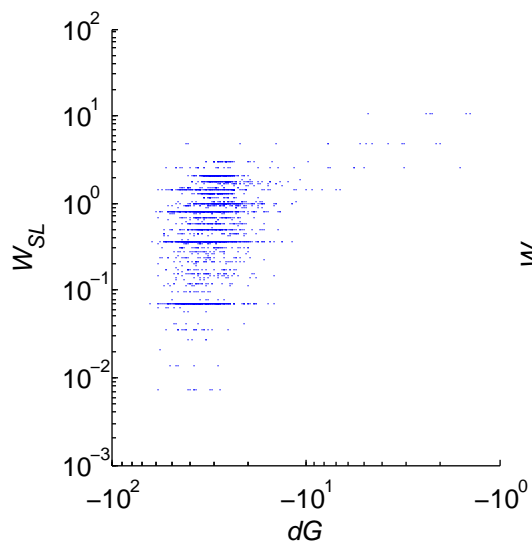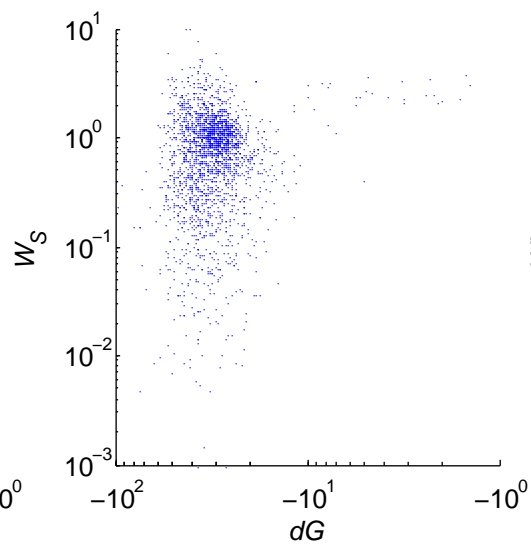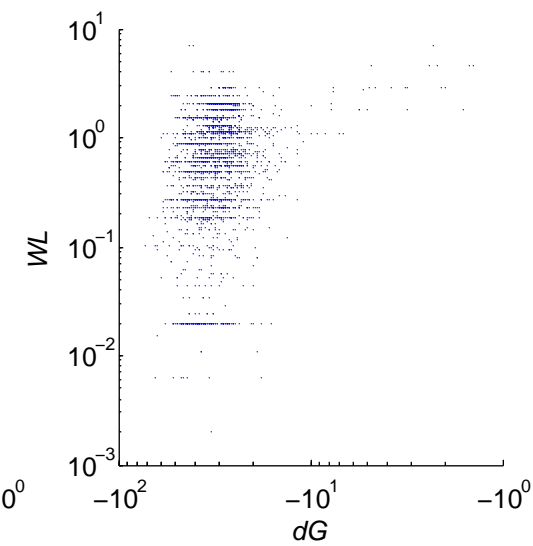

Supplement: Additional file 4 — Correlations between Wiener indices and the free energy of RNA. Correlations between Wiener indices and free energy for the dataset of 6,305 ncRNAs are shown. For convenience of visualization, both X and Y axes are scaled logarithmically. [file 1471-2105-9-188-S4.pdf]

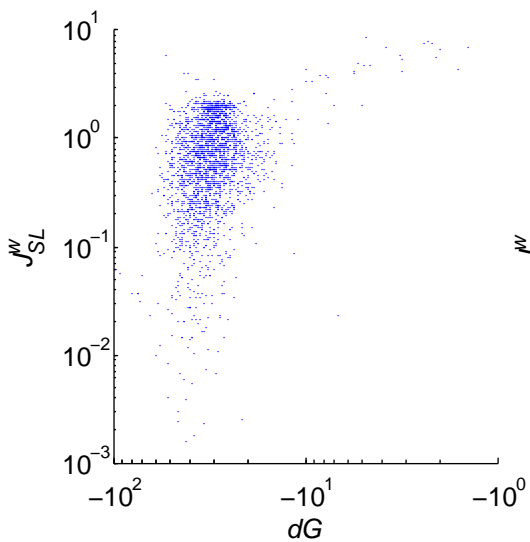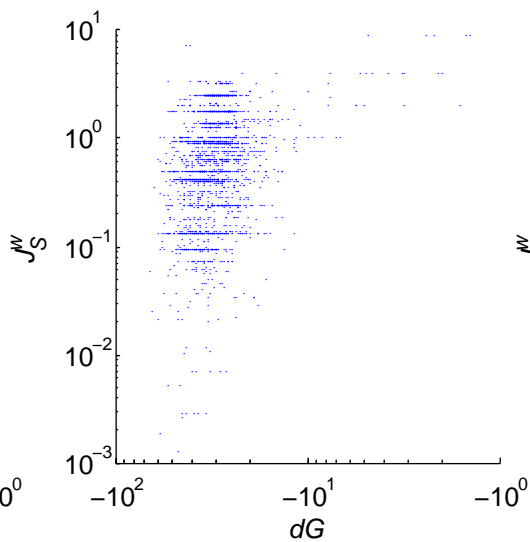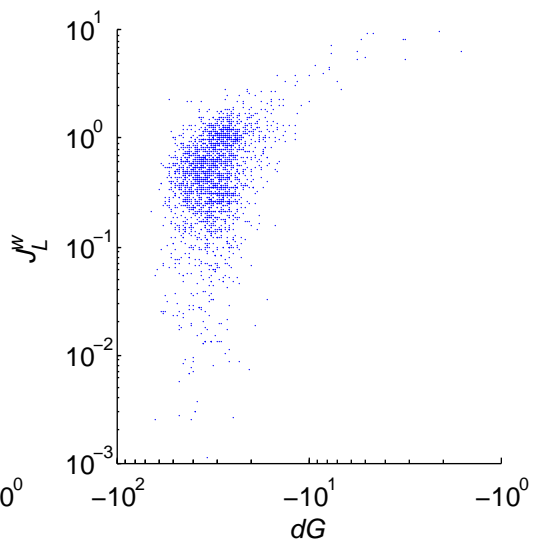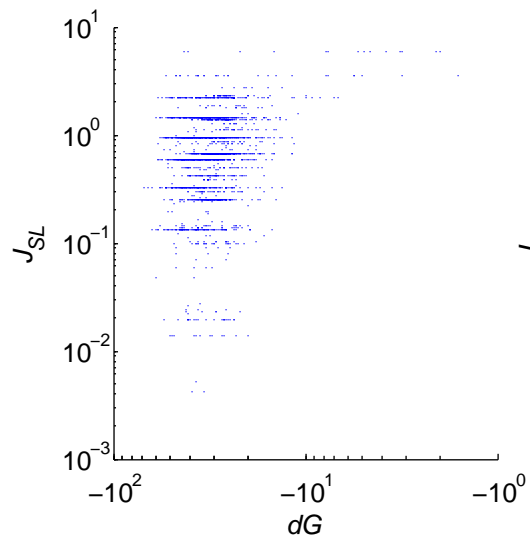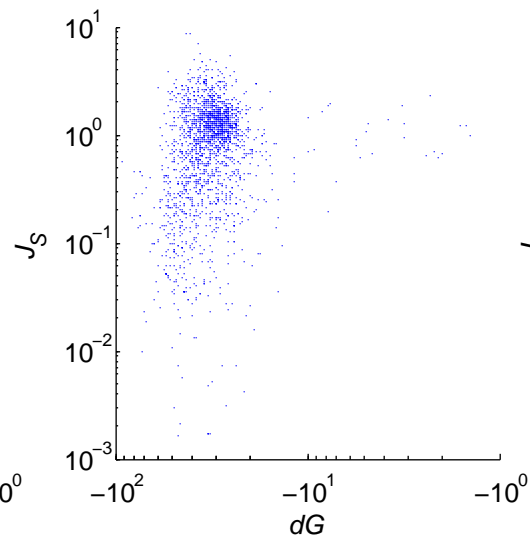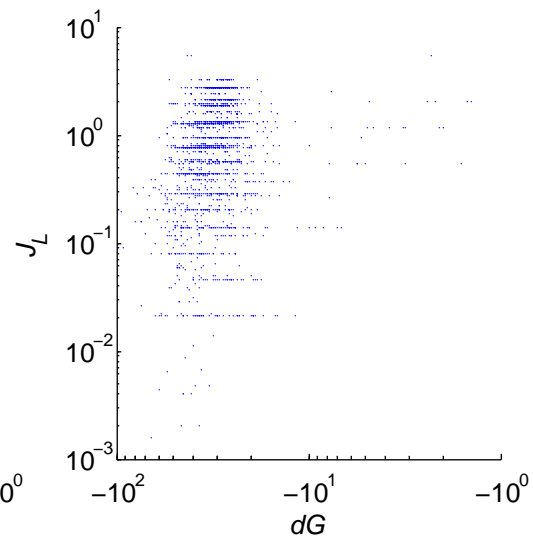

Supplement: Additional file 5 — Correlations between Balaban indices and the free energy of RNA. Correlations between Balaban indices and free energy for the dataset of 6,305 ncRNAs are shown. For convenience of visualization, both X and Y axes are scaled logarithmically. [file 1471-2105-9-188-S5.pdf]

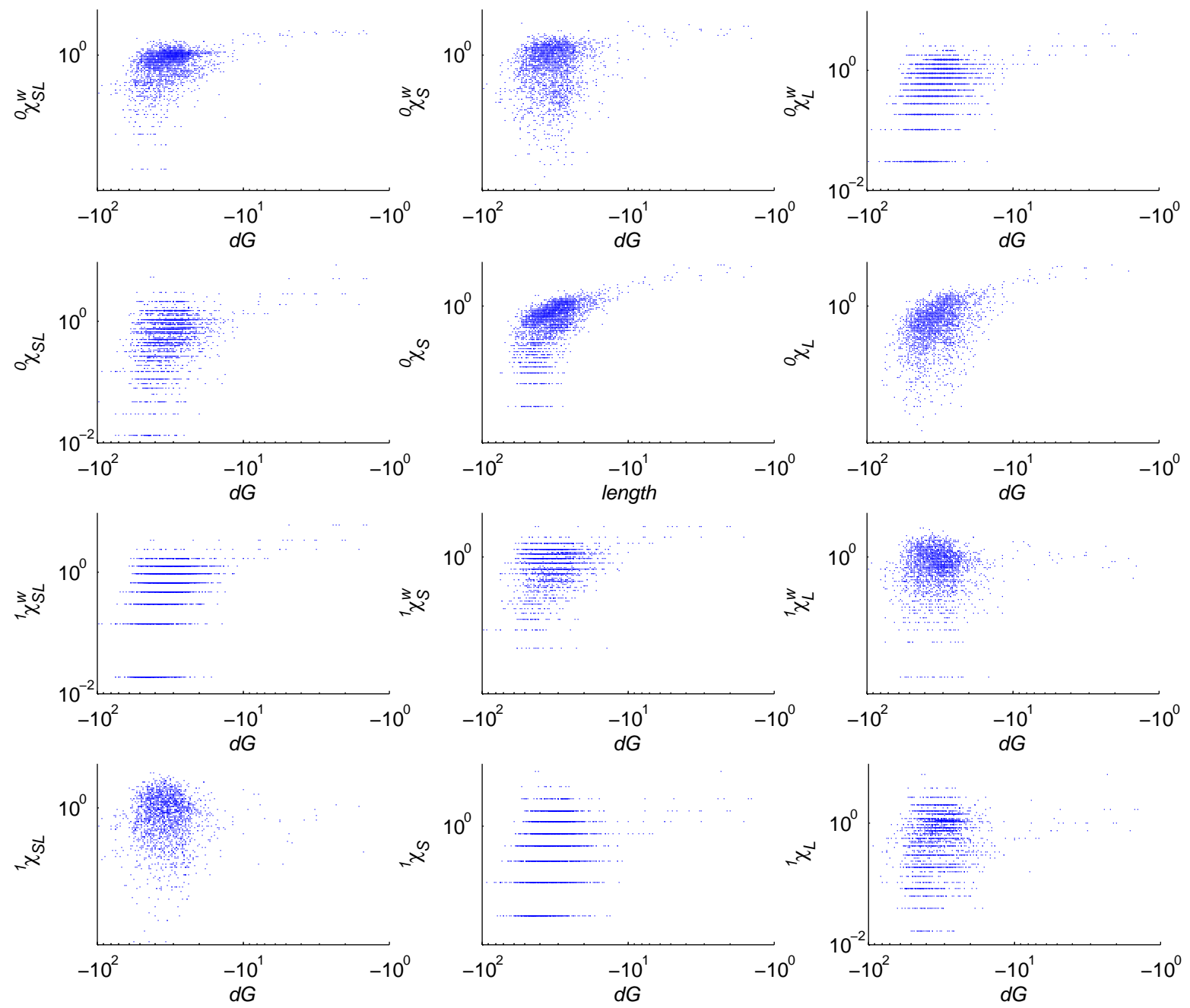

Supplement: Additional file 6 — Correlations between Randić indices and the free energy of RNA. Correlations between Randić indices and free energy for the dataset of 6,305 ncRNAs are shown. For convenience of visualization, both X and Y axes are scaled logarithmically. [file 1471-2105-9-188-S6.pdf]

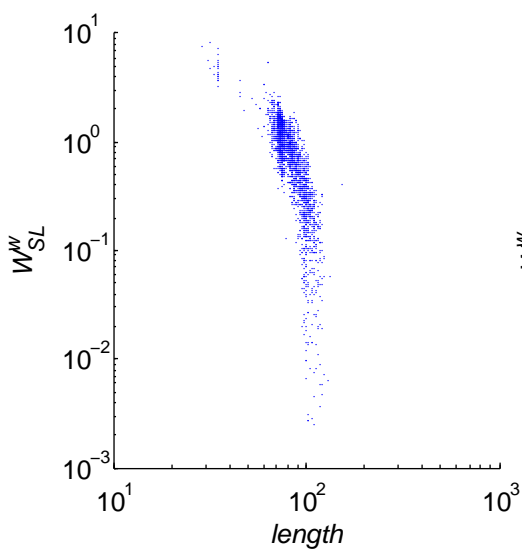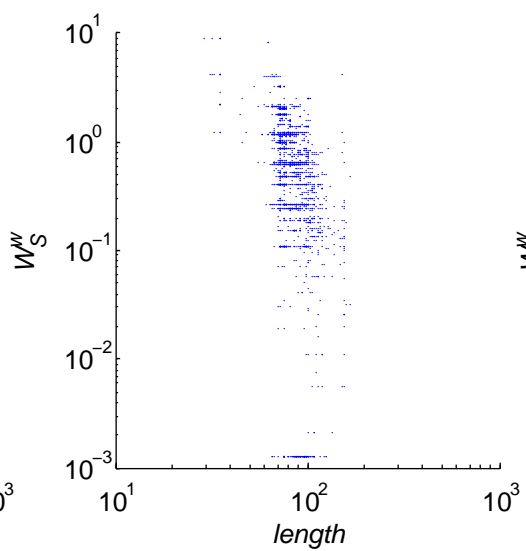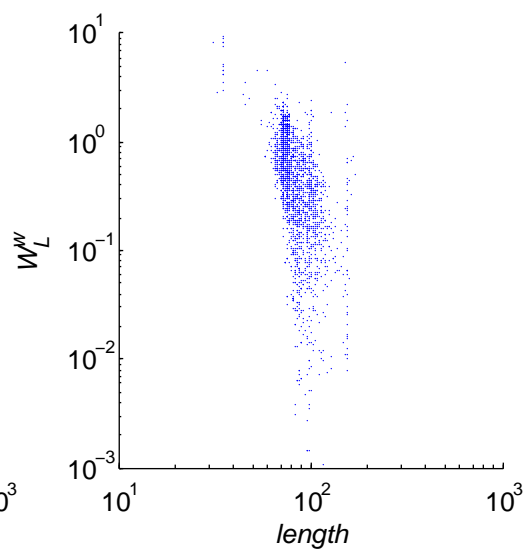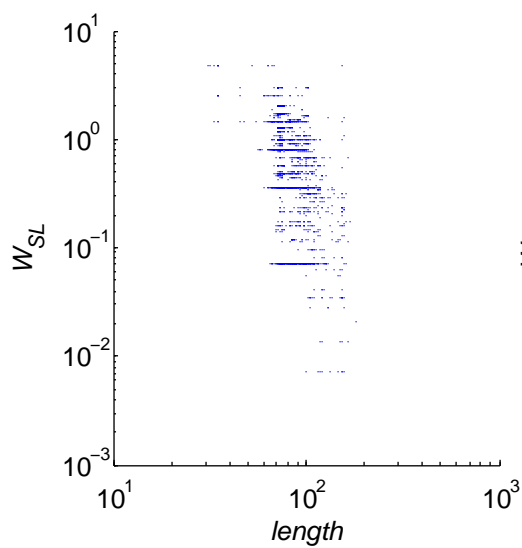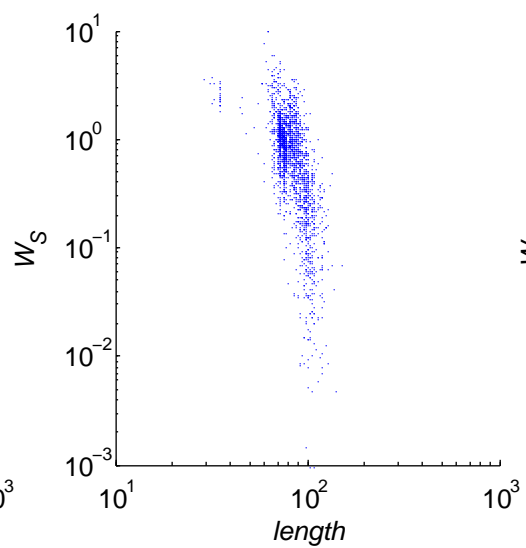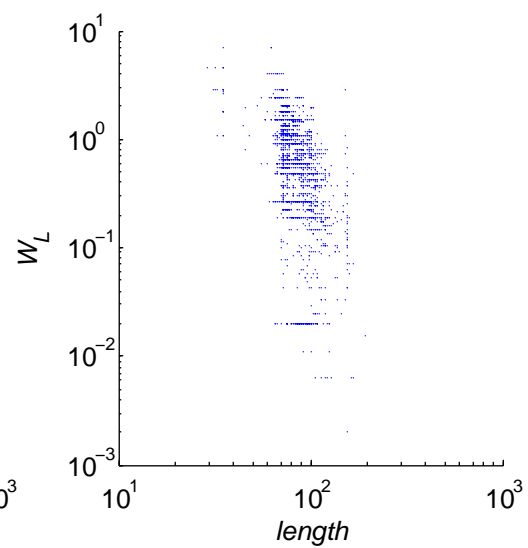

Supplement: Additional file 7 — Correlations between Wiener indices and the length of RNA. Correlations between Wiener indices and length for the dataset of 6,305 ncRNAs are shown. For convenience of visualization, both X and Y axes are scaled logarithmically. [file 1471-2105-9-188-S7.pdf]

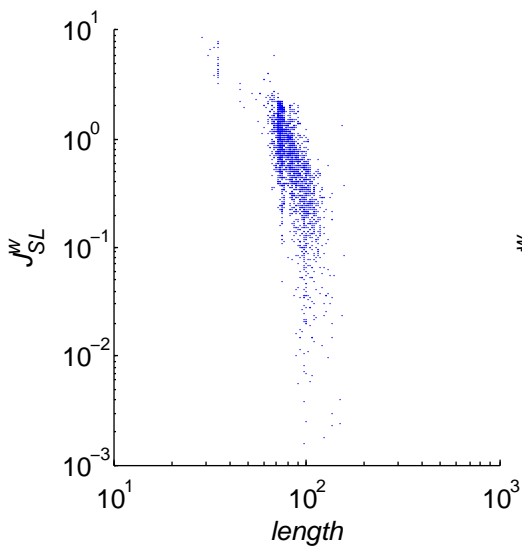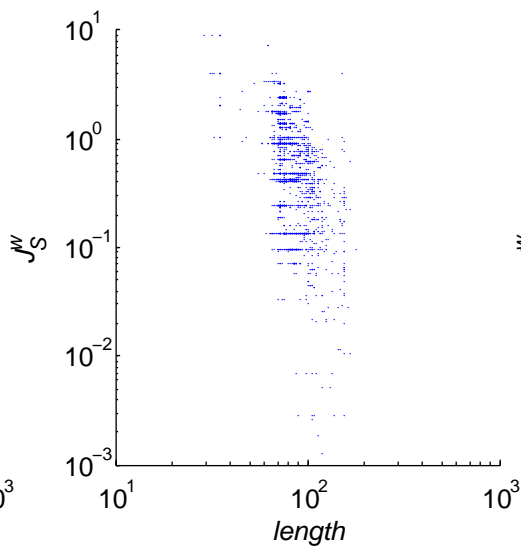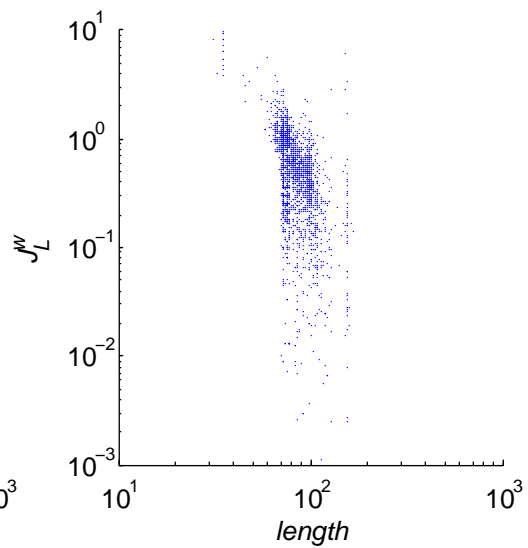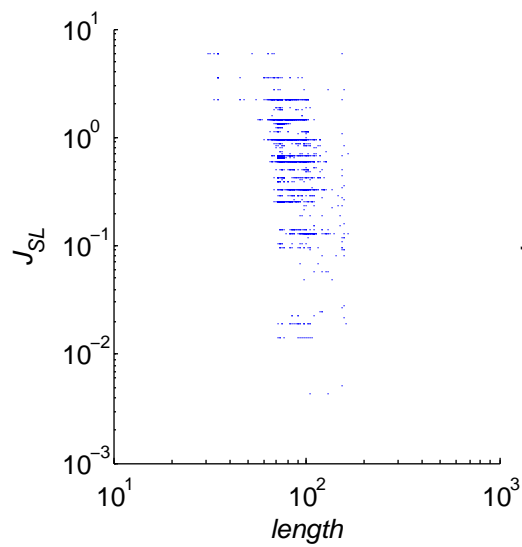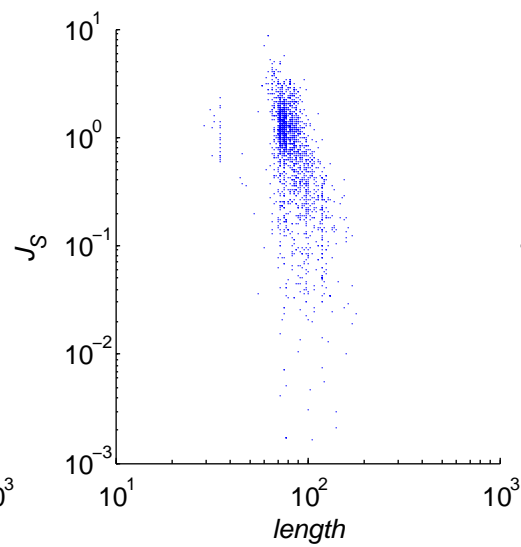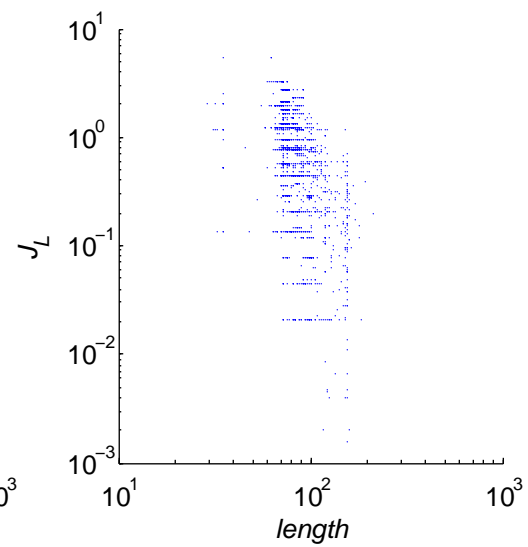

Supplement: Additional file 8 — Correlations between Balaban indices and the length of RNA. Correlations between Balaban indices and length for the dataset of 6,305 ncRNAs are shown. For convenience of visualization, both X and Y axes are scaled logarithmically. [file 1471-2105-9-188-S8.pdf]

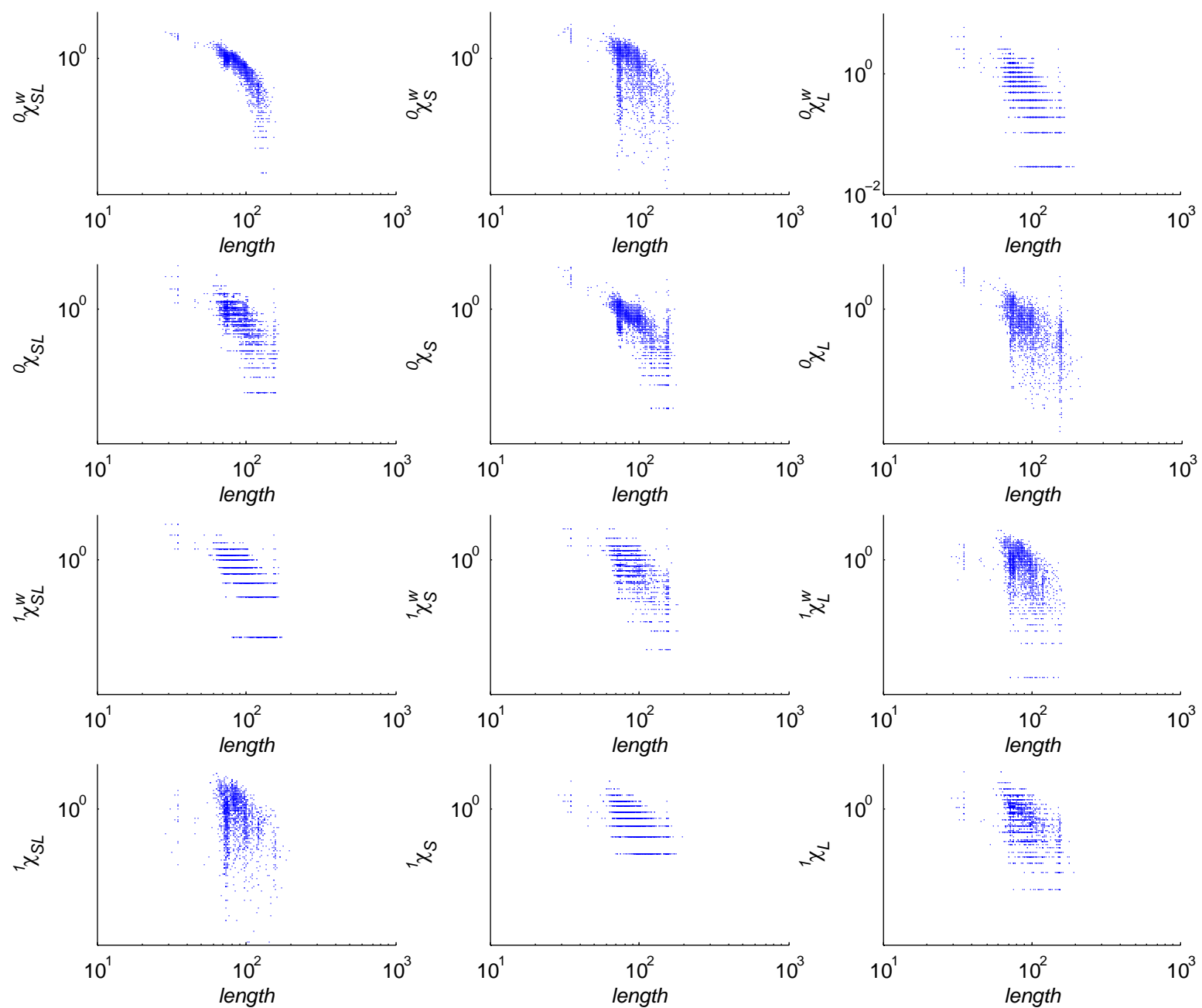

Supplement: Additional file 9 — Correlations between Randić indices and the length of RNA. Correlations between Randić indices and length for the dataset of 6,305 ncRNAs are shown. For convenience of visualization, both X and Y axes are scaled logarithmically. [file 1471-2105-9-188-S9.pdf]

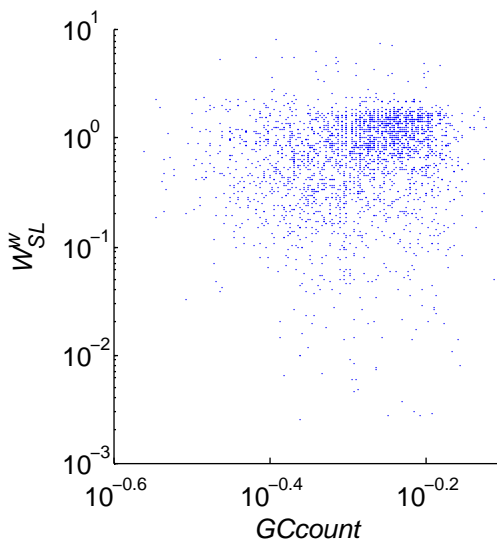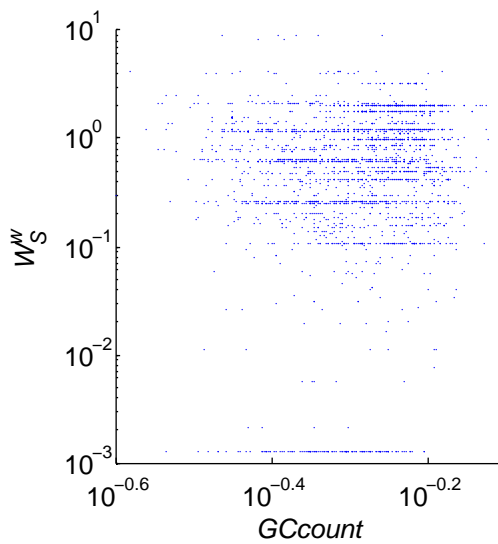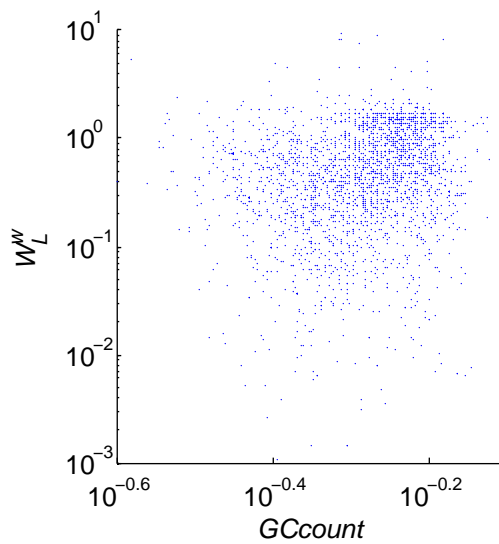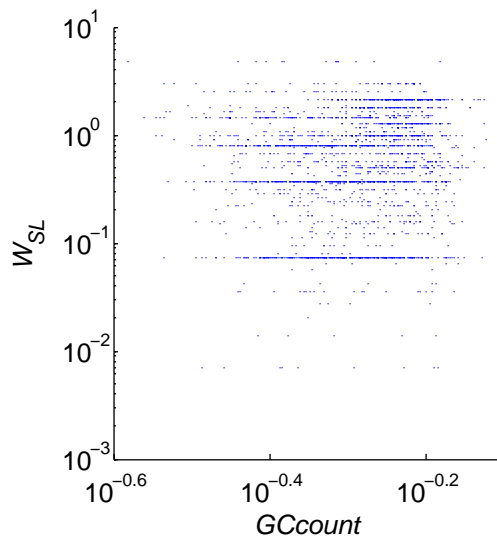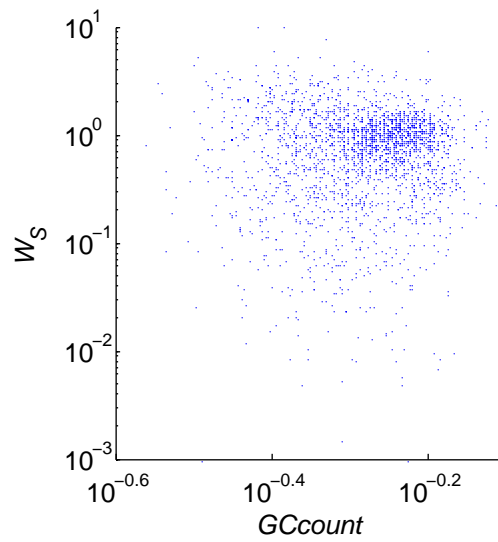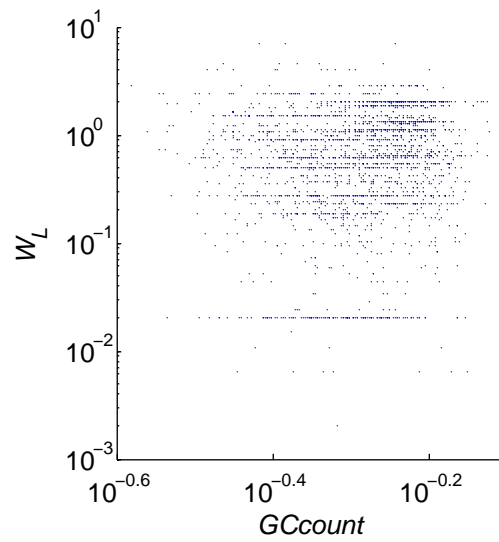

Supplement: Additional file 10 — Correlations between Wiener indices and the GC content of RNA. Correlations between Wiener indices and GC content for the dataset of 6,305 ncRNAs are shown. For convenience of visualization, both X and Y axes are scaled logarithmically. [file 1471-2105-9-188-S10.pdf]

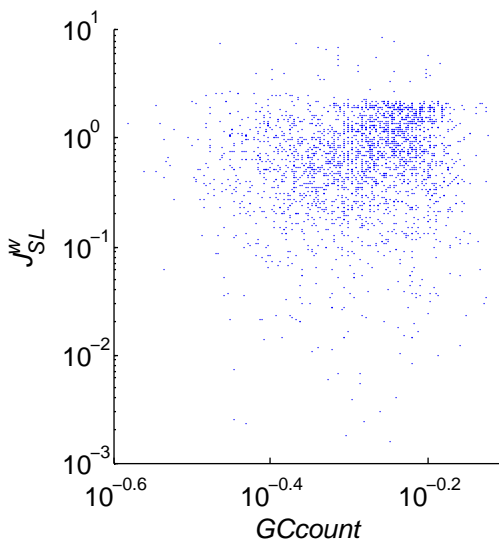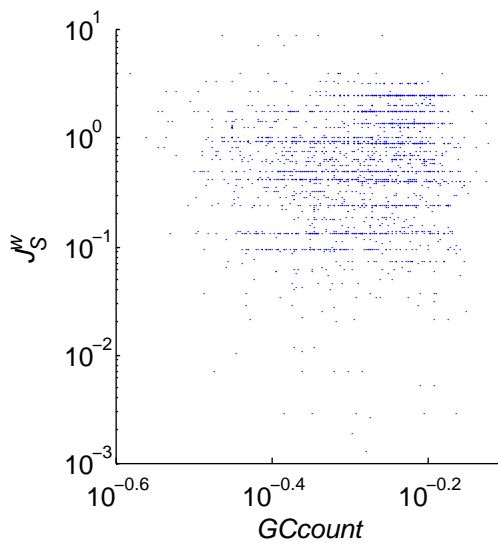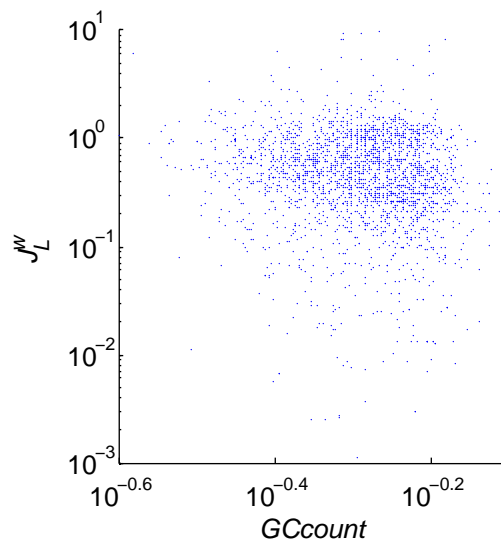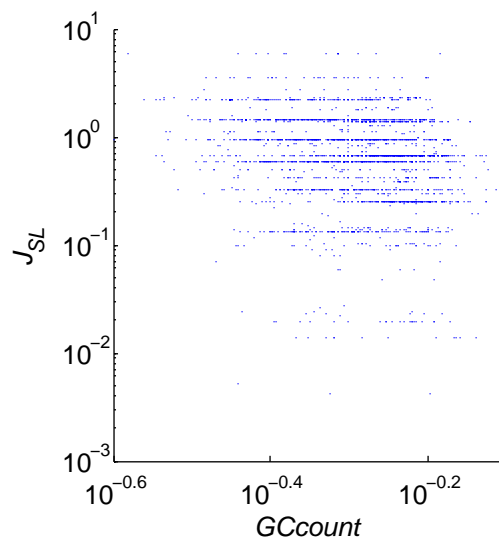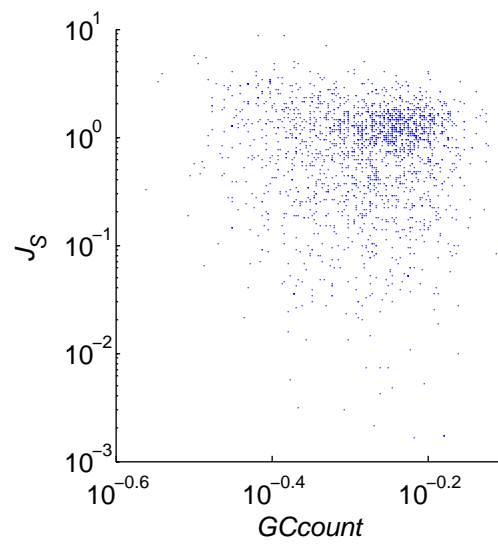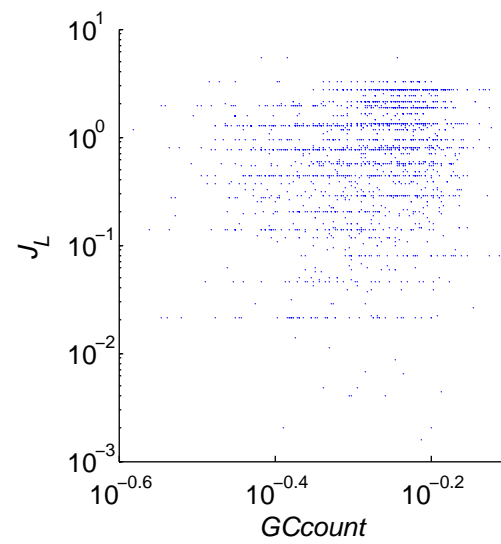

Supplement: Additional file 11 — Correlations between Balaban indices and the GC content of RNA. Correlations between Balaban indices and GC content for the dataset of 6,305 ncRNAs are shown. For convenience of visualization, both X and Y axes are scaled logarithmically. [file 1471-2105-9-188-S11.pdf]

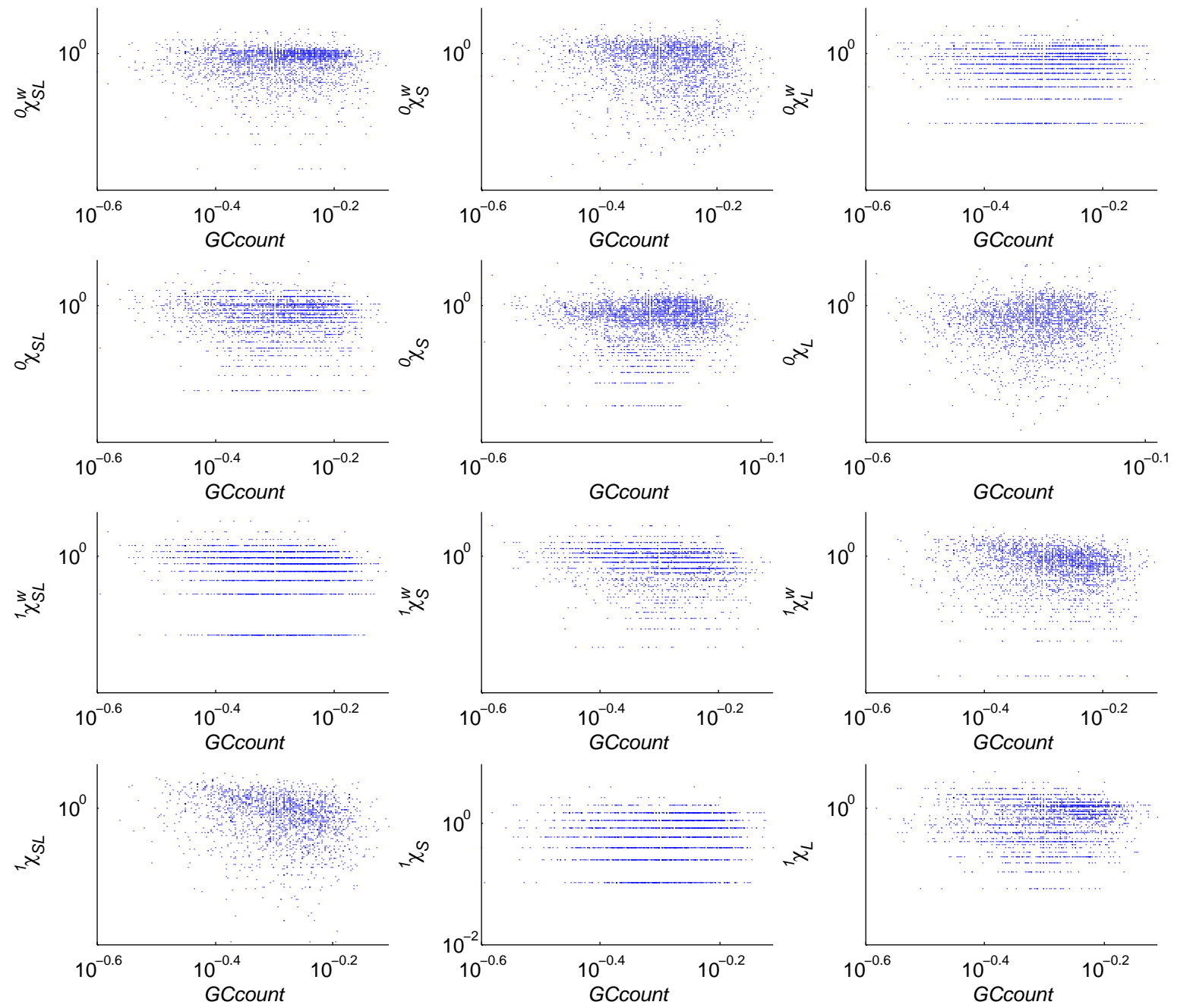

Supplement: Additional file 12 — Correlations between Randić indices and the GC content of RNA. Correlations between Randić indices and GC content for the dataset of 6,305 ncRNAs are shown. For convenience of visualization, both X and Y axes are scaled logarithmically. [file 1471-2105-9-188-S12.pdf]

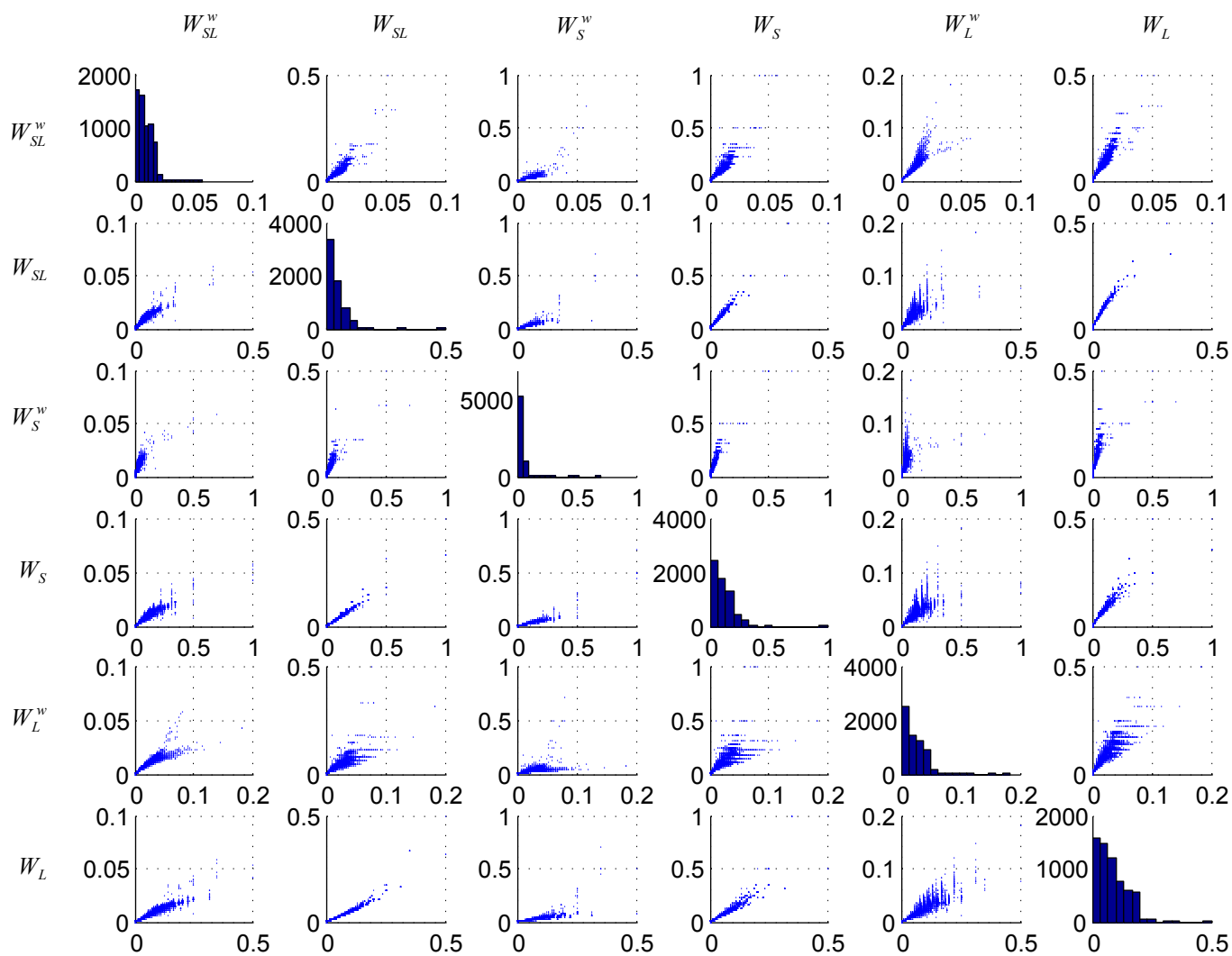

Supplement: Additional file 13 — Correlations between Wiener indices. Correlations between Wiener indices for the dataset of 6,305 ncRNAs are shown. The diagonal figures show the distributions of the Wiener indices. [file 1471-2105-9-188-S13.pdf]

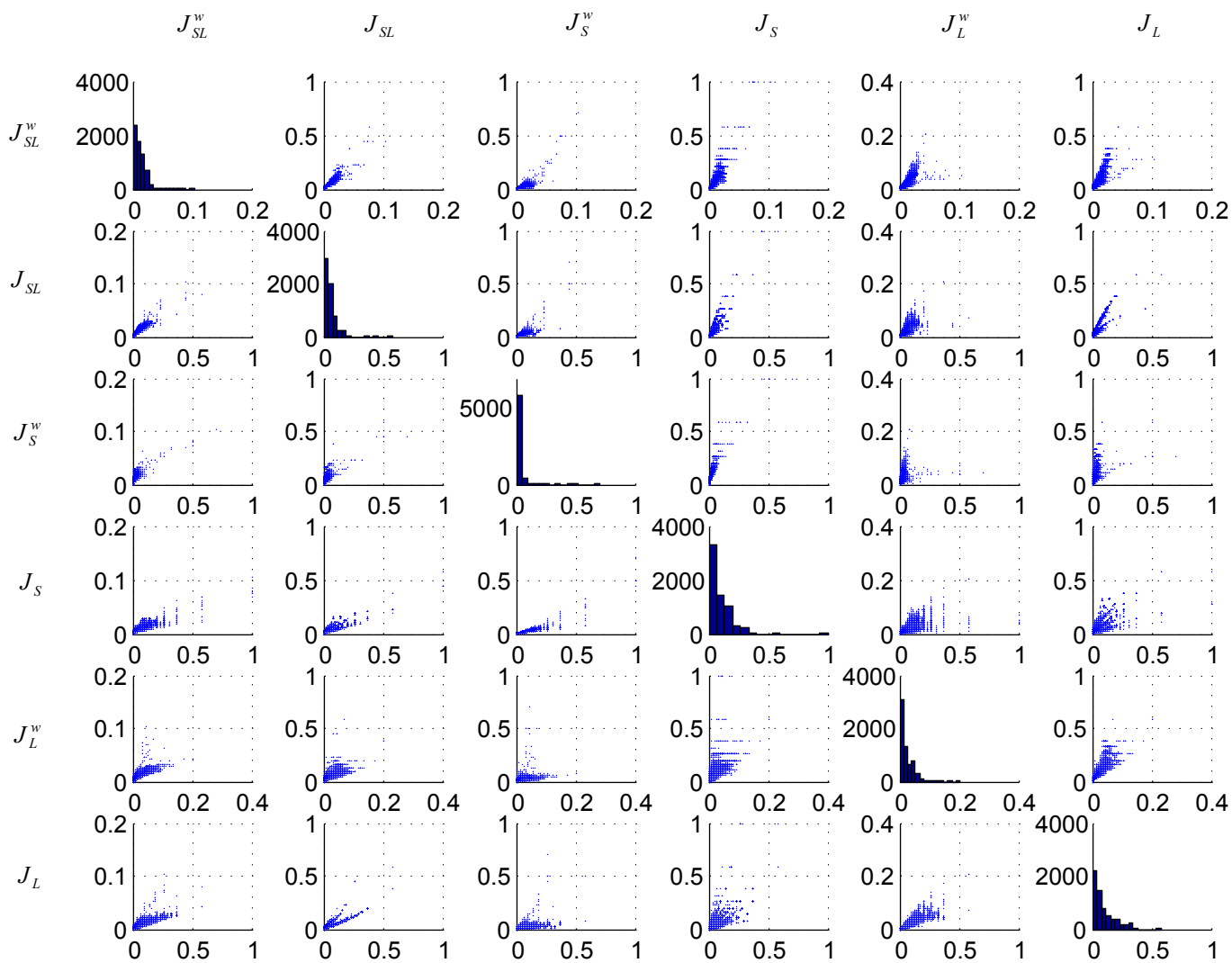

Supplement: Additional file 14 — Correlations between Balaban indices. Correlations between Balaban indices for the dataset of 6,305 ncRNAs are shown. The diagonal figures show the distributions of the Balaban indices. [file 1471-2105-9-188-S14.pdf]

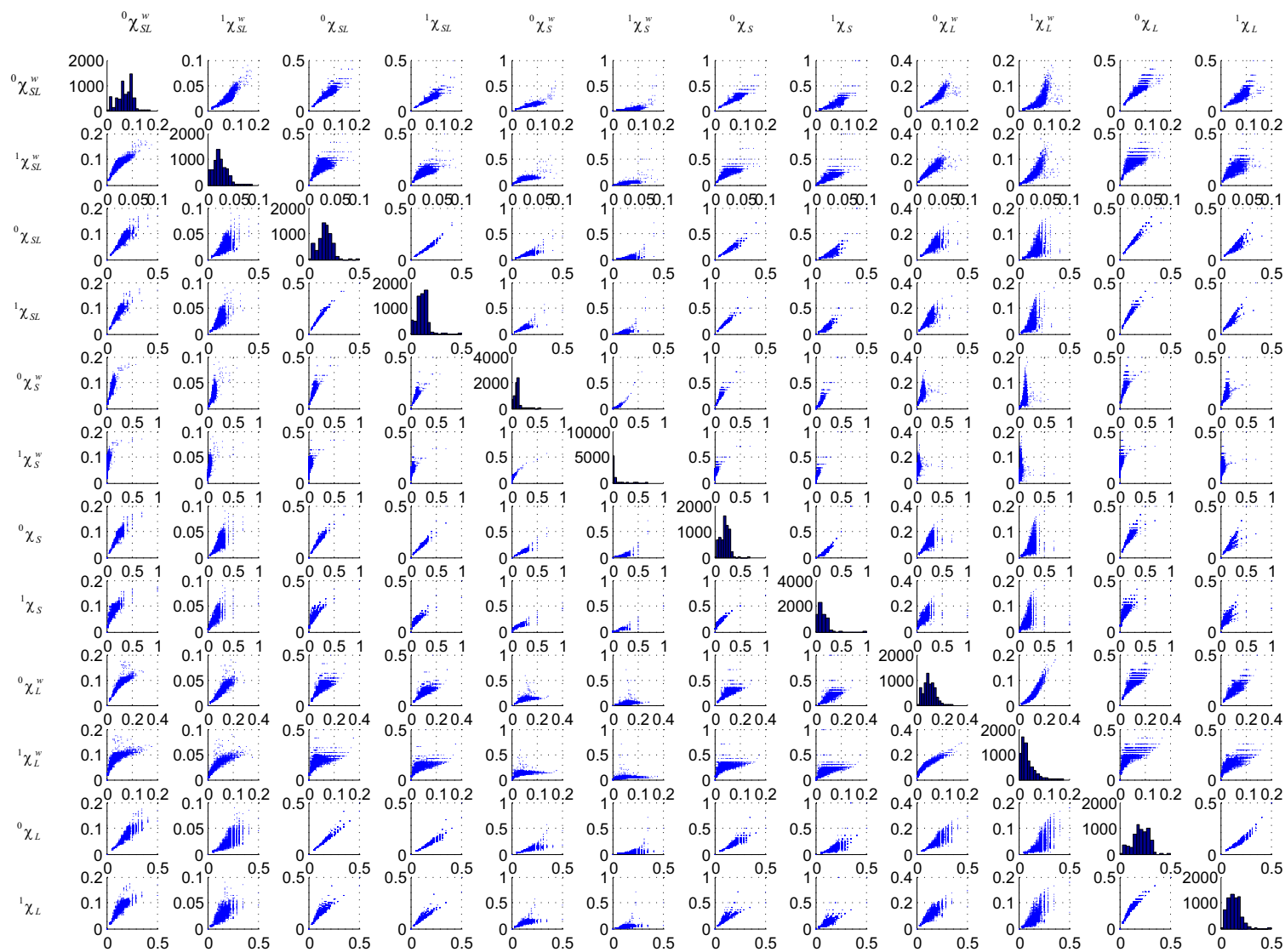

Supplement: Additional file 15 — Correlations between Randić indices. Correlations between Randić indices for the dataset of 6,305 ncRNAs are shown. The diagonal figures show the distributions of the Randić indices. [file 1471-2105-9-188-S15.pdf]
